# Aromaticity as a Guiding Concept for Spectroscopic Features and Nonlinear Optical Properties of Porphyrinoids

**DOI:** 10.3390/molecules23061333

**Published:** 2018-06-01

**Authors:** Tatiana Woller, Paul Geerlings, Frank De Proft, Benoît Champagne, Mercedes Alonso

**Affiliations:** 1 Eenheid Algemene Chemie (ALGC), Vrije Universiteit Brussel (VUB), Pleinlaan 2, 1050 Brussels, Belgium; Tatiana.Woller@vub.be (T.W.); pgeerlin@vub.be (P.G.); fdeprof@vub.be (F.D.P.); 2 Laboratoire de Chimie Théorique, Unité de Chimie Physique Théorique et Structurale, University of Namur, Rue de Bruxelles 61, B-5000 Namur, Belgium; benoit.champagne@unamur.be

**Keywords:** aromaticity, porphyrinoids, nonlinear optical properties, absorption spectra

## Abstract

With their versatile molecular topology and aromaticity, porphyrinoid systems combine remarkable chemistry with interesting photophysical properties and nonlinear optical properties. Hence, the field of application of porphyrinoids is very broad ranging from near-infrared dyes to opto-electronic materials. From previous experimental studies, aromaticity emerges as an important concept in determining the photophysical properties and two-photon absorption cross sections of porphyrinoids. Despite a considerable number of studies on porphyrinoids, few investigate the relationship between aromaticity, UV/vis absorption spectra and nonlinear properties. To assess such structure-property relationships, we performed a computational study focusing on a series of Hückel porphyrinoids to: (i) assess their (anti)aromatic character; (ii) determine the fingerprints of aromaticity on the UV/vis spectra; (iii) evaluate the role of aromaticity on the NLO properties. Using an extensive set of aromaticity descriptors based on energetic, magnetic, structural, reactivity and electronic criteria, the aromaticity of [4*n*+2] π-electron porphyrinoids was evidenced as was the antiaromaticity for [4*n*] π-electron systems. In agreement with previous studies, the absorption spectra of aromatic systems display more intense B and Q bands in comparison to their antiaromatic homologues. The nature of these absorption bands was analyzed in detail in terms of polarization, intensity, splitting and composition. Finally, quantities such as the average polarizability and its anisotropy were found to be larger in aromatic systems, whereas first and second hyperpolarizability are influenced by the interplay between aromaticity, planarity and molecular symmetry. To conclude, aromaticity dictates the photophysical properties in porphyrinoids, whereas it is not the only factor determining the magnitude of NLO properties.

## 1. Introduction

Porphyrinoids are macrocyclic compounds consisting of five-membered rings connected either directly or by bridging atoms, which usually exhibit a high degree of π-conjugation [1]. The constituent rings are pyrrole, furan, thiophene or other heterocyclic subunits, whereas the *meso* bridges are usually carbon atoms or linear chains thereof. As such, the term “porphyrinoid” covers a large number of compounds that share some distinctive structural features with the parent porphyrin, a tetrapyrrolic macrocycle with a *meso*-carbon bridge [2]. Among porphyrinoids, expanded porphyrins consisting of more than four pyrrole rings or related heterocyclic subunits have attracted considerable attention in the last decade due to their exceptional nonlinear optical properties, rich coordination properties and versatile aromaticity [3]. The extended π-conjugation system of expanded porphyrins enhances their conformational flexibility and they are ideal platforms to realize Möbius aromatic and antiaromatic species as well as figure-eight structures [4]. This huge structural diversity has led to a range of applications, including ion sensors [5], near-infrared (NIR) dyes [6], materials with two-photon absorption [7] and other nonlinear optical (NLO) properties [8] and molecular switches [9]. Given the tunable electronic, optical and photophysical properties of porphyrinoids, these systems constitute a promising building block for molecular optoelectronic devices [10].

Nowadays, expanded porphyrins are recognized as the test bed for investigating the correlation between molecular properties and (anti)aromaticity, since they provide congeneric macrocycles with [4*n*+2] and [4*n*] π-electrons that can be reversibly interconverted by two-electron redox reactions. This unique feature has been used to explore the relationship between aromaticity and various photophysical properties, including absorption and emission spectral features, excited-state lifetime and fluorescence [11]. Through these experimental studies, it is recognized that aromatic expanded porphyrins exhibit a very distinct absorption spectrum with intense and sharp B-like bands, also known as the Soret-like band, together with weak Q-like bands [12]. On the other hand, antiaromatic expanded porphyrins exhibit significantly attenuated absorption bands in the visible region with characteristic absorption tailing over the NIR region. These spectroscopic features are usually understood in terms of the symmetry and degeneracy of the frontiers molecular orbitals (MO) [13]. Moreover, excited-state dynamics of expanded porphyrins are dependent on their aromaticity, in such a way that larger excited-state lifetimes and fluorescence behavior is linked to aromatic structures [14]. In fact, the spectral changes measured upon excitation of the singlet ground state to the triplet-excited state in several expanded porphyrins proved experimentally Baird´s rule for the first time [15]. As such, expanded porphyrins provide the elusive “yin yang pair” to prove the reversal of Hückel (anti)aromaticity in the lowest triplet state [16].

In addition to spectroscopic features, aromaticity emerges as the key concept determining the NLO properties of porphyrinoids. For a series of closely related expanded porphyrins, a strong correlation between two-photon absorption (TPA) cross-sections values and the Nucleus-Independent Chemical Shift (NICS) values was found [14,17]. Specifically, compounds with greater aromatic character were found to give rise to higher TPA cross-section values, which is a third-order NLO process. Relative to the parent porphyrin, expanded porphyrins exhibit spectacularly high TPA cross-section values [7,18,19], so they are very promising candidates for biomedical applications, such as photodynamic therapy [20]. Moreover, several studies found a close relationship between molecular topology, aromaticity and NLO properties in expanded porphyrins [11,12] and demonstrate that Hückel-to-Möbius topological switches behave as efficient optical switches [21,22,23]. Besides NLO properties, our recent work highlights the importance of the macrocyclic aromaticity in determining the electron transport properties of expanded porphyrins; so redox-triggered aromaticity switches act as efficient conductance switching elements for molecular electronic devices [24].

From all these experimental and theoretical studies, aromaticity emerges as a guiding concept determining the electronic, photophysical and NLO properties of expanded porphyrins [11,25]. Nevertheless, aromaticity is not an observable and it lacks a rigorous definition [26,27,28]. Overall, aromatic species exhibit substantial energetic stabilization [29], low reactivity [30], bond-length equalization [31], highly negative nucleus-independent chemical shifts (NICS), exalted magnetic susceptibility, diatropic induced ring currents [32] and electron delocalization [33]. Its different manifestations of aromaticity have led to multiple ways for assessing the aromatic or antiaromatic character of molecules, resulting in a plethora of aromaticity indices based on energetic, reactivity structural, magnetic and electronic criteria. Accordingly, most of the authors advise to use a set of aromaticity descriptors based on different physical properties to characterize (anti)aromatic compounds [27,34,35]. When using a large set of aromaticity indicators, conclusions regarding the aromaticity are much reliable, especially if all criteria provide the same trends. Recently, the aromaticity of porphyrinoids was shown to be particularly “multifaceted”, resulting in large discrepancies between energetic and magnetic descriptors [36]. Thus, the quantification of aromaticity in porphyrinoids remains challenging.

In our recent computational research on expanded porphyrins, we have proposed a set of descriptors for measuring the global aromaticity of porphyrinoids based on energetic, structural, magnetic and reactivity criteria [37,38,39]. Although some discrepancies exist between the different indices, overall the energetic, reactivity, structural, magnetic and electronic indices nicely agree on the general (anti)aromatic features of these compounds in agreement with the annulene model [40,41]. Nowadays, the annulene model is widely used to predict qualitatively the aromaticity of porphyrinoids [42,43,44]. According to this model, the ring system of a porphyrinoid is treated as a bridged heteroannulene, so aromaticity can be predicted in a qualitative way by formally applying the [4*n*+2] Hückel rule to the annulene substructure [45,46]. In the case of all-pyrrole porphyrinoids containing no confused rings, the annulene-type conjugation pathway (CP) passes through the iminic nitrogens and bypasses the amino NH groups [47]. Nevertheless, these macrocycles sustain multiple conjugation pathways and, in principle, Hückel´s rule should not be applied to polycyclic π-systems. By this reason, we have investigated the conjugated pathways in porphyrinoid compounds using several local aromaticity indicators [48], including the recently introduced AV1245 [49] and AV_min_ [50] electronic indices. All indices agree on the general features of these compounds such as the fulfilment of Hückel´s rule, but only AV_min_ is capable of recognizing the annulene pathway as the most aromatic one in all studied porphyrinoids. A careful examination of our results shows that some indices overestimate the aromaticity in the pathways because they are based on average values and, therefore, conceal disconnected fragments that are responsible for the loss of aromaticity.

**Figure 1:**
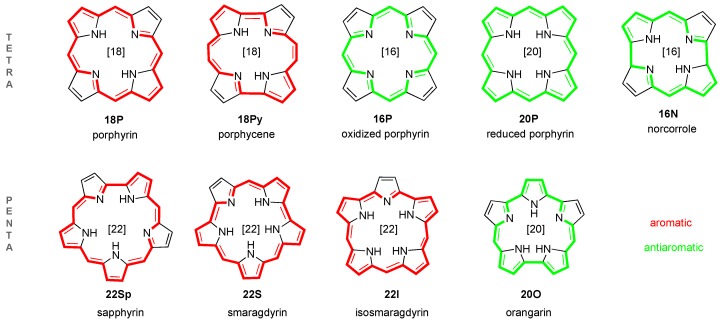
Selected Hückel porphyrinoids and their expected aromaticity according to the annulene model. The annulene-type conjugation pathway is depicted with bold colored bonds.

**Figure 2:**
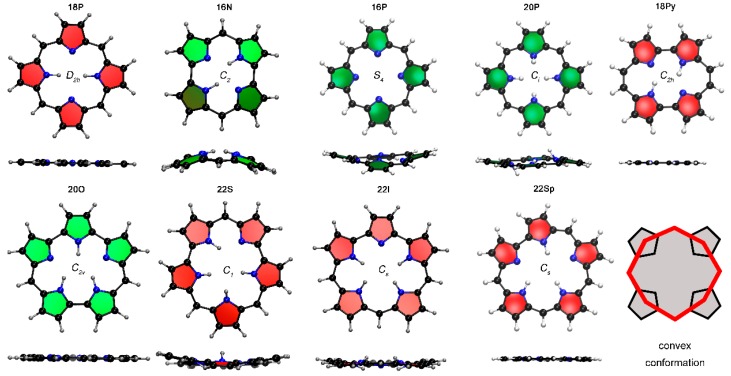
Hückel conformations computed for different porphyrinoids (top and side views). Red five-membered rings are linked to aromatic structures, whereas green ones to antiaromatic configurations. The symmetry point group together with a schematic representation of the convex conformer are also shown.

Herein, we examine the aromaticity of Hückel porphyrinoids with varying number of π-electrons (Figure 1). The selected porphyrinoids involves tetra- and pentapyrrolic macrocycles in which the preferred conformation is expected to be a convex conformation with all inward-pointing pyrrole subunits [1]. While the polygon closed by the smallest macrocyclic circuit is actually concave, the polygon formed by *meso* positions and centroids of cyclic subunits is convex (Figure 2). Among tetraphyrins, the archetypal porphyrin (**18P**) and porphycene (**18Py**) are considered together with the unstable norcorrole (**16N**). Besides, the doubly oxidized [51] and reduced [52] congeners of the regular porphyrin are also investigated with 16 π- and 20 π-electronic structures (**16P** and **20P**), respectively. Among pentapyrrolic macrocycles, orangarin (**20O**), smaragdyrin (**22S**) and isosmaragdyrin (**22I**) were selected since the free curvature values are smaller than that of porphyrins and consequently yield only convex conformations that are nearly planar [1]. Additionally, sapphyrin with four *meso*-carbon bridges (**22Sp**) was also included in the test set since this is one of the most extensively investigated expanded porphyrins from the experimental point of view [53]. However, the conformational preferences of sapphyrins do not follow a simple pattern, and two conformations have been observed experimentally, corresponding to a convex and a concave structure, the later containing an inverted pyrrole ring. The relative stability of these conformers depends on peripheral substitution, core modifications, and protonation state, among others [1,54]. Porphyrinoids with large conformational flexibility such as regular pentaphyrins and hexaphyrins are not considered here since Hückel, Möbius and twisted-Hückel topologies are feasible depending on the conditions and substitution.

First, the aromaticity of the selected Hückel porphyrinoids has been quantified using a large set of aromaticity descriptors based on energetic, magnetic, structural, reactivity and electronic criteria. As such, the validity of the annulene model and the performance of the different indices to describe Hückel aromaticity in prototypical porphyrinoids are first assessed in detail. Secondly, we investigate the fingerprints of (anti)aromaticity on the spectroscopic features, particularly on the absorption spectra. Finally, motivated by the invoked influence of the aromaticity on the TPA cross-section values [14,17], the structure–property relationship between aromaticity and nonlinear optical properties have been established for these porphyrinoids. Our main aim is to assess the role of aromaticity in determining the photophysical and NLO properties of Hückel porphyrinoids.

## 2. Results and Discussion

### 2.1. Structure-Aromaticity Relationship in Hückel Porphyrinoids

The three-dimensional structures of the most stable conformations of porphyrin (**18P**), porphycene (**18Py**), oxidized porphyrin (**16P**), reduced porphyrin (**20P**), norcorrole (**16N**), sapphyrin (**22Sp**), orangarin (**20O**), smaragdryin (**22S**) and isosmaragdyrin (**22I**) are shown in Figure 2. The symmetry point group of the different structures is *D*_2h_ for **18P**, *C*_2*h*_ for **18Py**, *C*_2*v*_ for **20O**, *C*_2_ for **16N**, *C_s_* for **22I** and **22Sp** and *C*_1_ for **22S**. Upon two-electron redox reactions, the symmetry point group of the porphyrin changes to an *S*_4_ structure for **16P** and *C_i_* for **20P**. In all the porphyrinoids, a Hückel untwisted topology is preferred with no inverted pyrrole rings. In the case of the sapphyrin (**22Sp**), two different conformations were considered, as well different tautomers for each conformer (Figure S1). According to our relative energies, the convex structure with no inverted pyrrole rings is the most stable for the unsubstituted neutral sapphyrin and the inversion of a pyrrole ring increases by ca. 10 kcal mol^−1^ the energy. The convex conformations of **18P**, **18Py**, **20O** and **22Sp** are totally planar and ring-strain free (Φ_p_ = 0 and *Π* = 1), while the structures of **16N** and **16P** are highly distorted from planarity as indicated by the torsional strain descriptors Φ_p_ and *Π* (Table 1). The smallest porphyrinoid included in our set **16N**, containing just two *meso*-bridges, increases the torsional ring strain and precludes an effective π-conjugation (Φ_p_ = 31.7 and *Π* = 0.49), what can be linked to the instability of this macrocycle. For the pentapyrrolic macrocycles, all the energy minima are relatively planar with an efficient π-conjugation, as denoted by the low Φ_p_ and large π-conjugation index (*Π*). Since the number of π-electrons along the annulene-type conjugation pathway is different, the selected porphyrinoids provide an optimum test bed for investigating the correlation between (anti)aromaticity and nonlinear optical properties and spectroscopic features.

To provide further insight into the structure-aromaticity relationship in Hückel porphyrinoids, several aromaticity descriptors based on distinct criteria were computed for the different systems. The degree of global aromaticity in tetrapyrroles (**18P**, **18Py**, **16P**, **20P** and **16N**) and pentapyrroles (**20O**, **22S**, **22I** and **22Sp**) has been quantified using reactivity (Δ*η*) and magnetic [*Λ*, NICS(0), NICS(1) and NICS_zz_(1)] descriptors and they are collected in Table 1, together with the torsional descriptors (*Π* and Φ_p_) and the HOMO-LUMO energy gap (ΔE_H-L_). Each of these aromaticity descriptors has their advantages and limitations [35,41], so it is advisable to use a set of indices based on different physicochemical properties to characterize (anti)aromatic compounds. When a large set of aromaticity indicators is used, conclusions are much more reliable, especially when the different criteria agree on the (anti)aromatic character for the set of compounds analyzed [41].

In agreement with the annulene model, all the Hückel porphyrinoids with [4*n*+2] π-electrons (**18P**, **18Py**, **22S**, **22Sp** and **22I**) are highly aromatic, with positive Δ*η* values, highly negative *Λ* and NICS values as well as bond-delocalized structures with HOMA values close to 0.9. By contrast, **16P**, **20P**, **16N** and **20O** are antiaromatic with negative Δ*η* values and paratropic ring currents, as denoted by the positive NICS-based indices and *Λ.* From all the aromaticity descriptors, the energetic index is the less reliable. The uncorrected ISE values are positive (Table S1) for all Hückel porphyrinoids, which is mainly due to the unbalanced *syn*-*anti* diene conformations at both sides of the reaction scheme. As such, the application of *syn*-*anti* corrections is needed for the evaluation of the energetic descriptor, which are evaluated as the energy difference between the dihydrogen derivative of the *meso*-methyl octaphyrin and its respective nonaromatic isomer [55]. However, most of the dihydrogen derivatives of the methylene isomers suffer drastic structural changes during the optimization step, resulting in biased ISE_corr_ values. By this reason, **16N** and **20O** exhibits a positive ISE_corr_ values despite its antiaromaticity. By contrast, the evaluation of the *Λ* and Δ*η* does not require *syn*/*anti* corrections, so the isomerization method is a more effective approach for evaluating the exaltation and relative hardness of porphyrinoids. As such, **16N** and **20O** exhibit negative relative hardness and positive exaltation in full agreement with the positive NICS-based indices.

Besides energetic, magnetic and structural descriptors, the recently-developed electronic indices for large circuits [AV1245 and AV_min_] have been evaluated (Table 1). AV1245 index measures the average delocalization in bonds 1–2 and 4–5 along a ring of more than six members [49]. The four-center multicenter indices (including atoms in positions 1, 2, 4 and 5) of each consecutive five-atom fragment in the circuit are averaged out in the AV1245 index. In our recent study, AV_min_, the minimal absolute value of the aforementioned four-center MCI values along the ring, was shown to be even a better electronic descriptor of aromaticity since it is not based on average values [48]. Both indices do not rely on reference values and they do not suffer from large numerical precision errors. As can be inferred from the electronic indices in Table 1, aromatic porphyrinoids (**18P**, **18Py**, **22Sp**, **22S** and **22I**) exhibit values of AV1245 larger than 2, together with large AV_min_ values. By contrast, antiaromatic **16P**, **20P**, **16N** and **20O** present low values for both indices, supporting that the extent of transferability of the delocalized electrons between bonds 1–2 and 4–5 is small, as expected in antiaromatic systems. In addition, from the ΔE_H-L_ values, it is clear that aromatic porphyrinoids exhibit significantly larger HOMO-LUMO gap than antiaromatic porphyrinoids, in agreement with previous studies [8,13,24].

**Figure 3:**
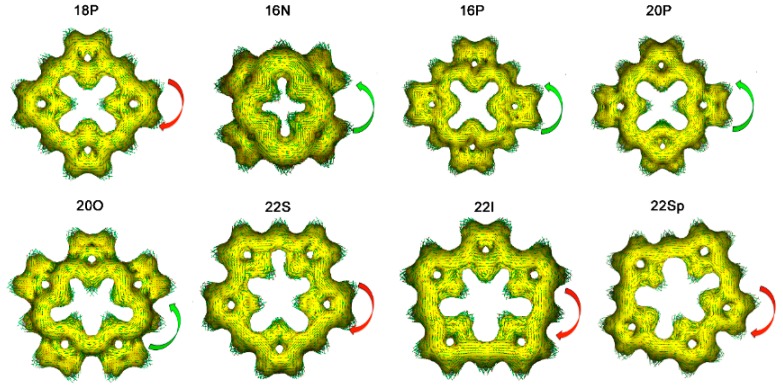
AICD plots of Hückel porphyrinoids. The large arrow denotes the direction of the induced ring current: clockwise for diatropic ring currents and anticlockwise for paratropic ring currents (isosurface value 0.03 a.u.).

To further assess the (anti)aromaticity of the different Hückel porphyrinoids, the so-called AICD (anisotropy of the induced current density) plots were evaluated (Figure 3 and Figure S2) [56]. These plots display the density and the direction of the induced ring current when an external magnetic field is applied, usually perpendicularly to the π-system [57]. To distinguish between diatropic and paratropic ring currents, the current density vectors are plotted onto the AICD isosurface. From these plots, it is clear that aromatic species (**18P**, **18Py**, **22S**, **22Sp** and **22I**) display diatropic ring currents (clockwise current density vectors), while antiaromatic porphyrinoids (**16P**, **20P**, **16N** and **20O**) display paratropic ring currents (anticlockwise current density vectors). Therefore, there is a strong relationship between the number of π-electrons on the annulene-type conjugation pathway and the aromaticity of our selected porphyrinoids. 

**Figure 4:**
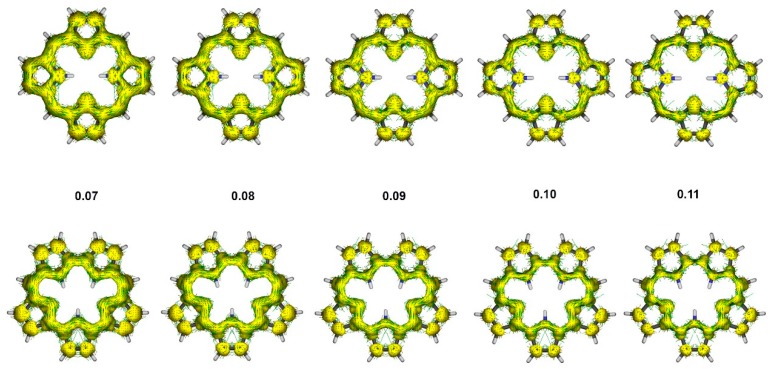
AICD plots of (18)porphyrin (**top**) and (20)orangarin (**bottom**) for different isosurface values (in a.u.).

Beside the direction of the current density vectors, the AICD plot of aromatic and antiaromatic systems differ in global shape and in its evolution upon increasing the isosurface values. For instance, the AICD plot of **16N** and **20O** are characterized by a significantly higher density in the inner circuit than their aromatic counterparts. This trend is enhanced upon increasing gradually the isovalue from 0.03 a.u. to 0.11 a.u. Until an isovalue of 0.09 a.u., the annulene pathway is still continuous for **18P** (Figure 4), whereas the AICD surface of the inner circuit passing through the pyrrole ring starts to break for an isovalue equal to 0.07 a.u. By contrast, the AICD plot of **20O** going through the annulene pathway is already discontinuous at 0.07 a.u. while the inner circuit is still intact at 0.11 a.u.

### 2.2. Photophysical Properties of Porphyrinoids

**Figure 5:**
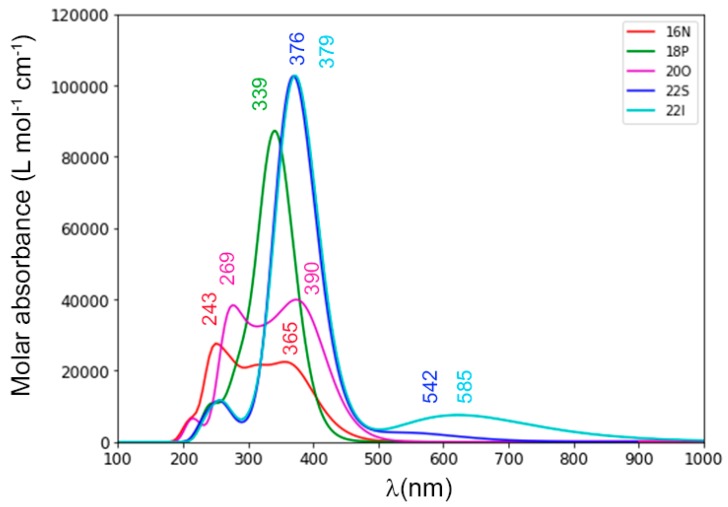
UV/vis absorption spectra of unsubstituted Hückel porphyrinoids simulated using Gaussian functions with a halfwidth of 2686 cm^−1^.

In the discussion of the photophysical properties, first the UV/vis spectra of **16N**, **18P**, **20O**, **22S** and **22I** are discussed and the fingerprints of (anti)aromaticity of the absorption spectra are elucidated. Then, our findings are validated for the other set of Hückel porphyrinoids, namely **16P**, **20P**, **18Py** and **22Sp**. Table 2, Table 3, Table 4, Table 5 and Table 6 collect the characteristic properties of the first five electronic transitions and the most intense electronic transitions of porphyrinoids **16N**, **18P**, **20O**, **22S** and **22I**. As can be seen in Figure 5, the absorption spectrum of aromatic macrocycles consists of intense and sharp peaks, while broader and weaker absorption bands are found in the absorption spectrum of antiaromatic systems. For the most intense absorption band, we observe the following order according to the molar extinction coefficient, which is related to the oscillator strength (*f*_osc_) of the transition: **22I** ≥ **22S** > **18P** > **20O** > **16N**. As expected, the absorption bands become more intense for larger conjugation pathways in the selected porphyrinoids. Nevertheless, the effect of larger conjugation pathway is less important than the aromaticity or antiaromaticity of the π-conjugated system.

In aromatic porphyrinoids, the most intense absorption, namely the Soret or B band, corresponds to electronic transitions involving H, H−1, L and L+1 orbitals (Table 3, Table 5 and Table 6). By contrast, the main electronic transitions in antiaromatic systems correspond to high-energy transitions (Table 2 and Table 4) [58]. Nevertheless, the first high-intensity band of **20O** is dominantly of HOMO−1→LUMO+1 character. In addition, the absorption spectrum of aromatic macrocycles displays very weak absorption bands in near-infrared (IR) region, the so-called Q bands. As such, the absorption spectra of aromatic systems are characterized by the presence of weak Q bands (*λ* > 500 nm) and very intense B bands (300 nm < *λ* < 400 nm and *f*_osc_ > 0.9). In comparison to **22S** and **22I**, the oscillator strength associated to the Q band in **18P** is particularly small. By contrast, the B bands and the absorption bands associated to high-energy electronic transitions are significantly weaker (*f*_osc_ < 0.9) in the absorption spectra of antiaromatic systems and the Q bands (*f*_osc_ < 0.008) are either nonexistent or too weak to be measured. Aside (20)orangarin, the most intense absorption band of aromatic macrocycles are red-shifted with respect to those of antiaromatic systems. Consequently, the overall features of all absorption spectra in those macrocycles demonstrate a close structure-property relationship between (anti)aromaticity and absorption, in excellent agreement with the experimental observations [11,12,13].

**Figure 6:**
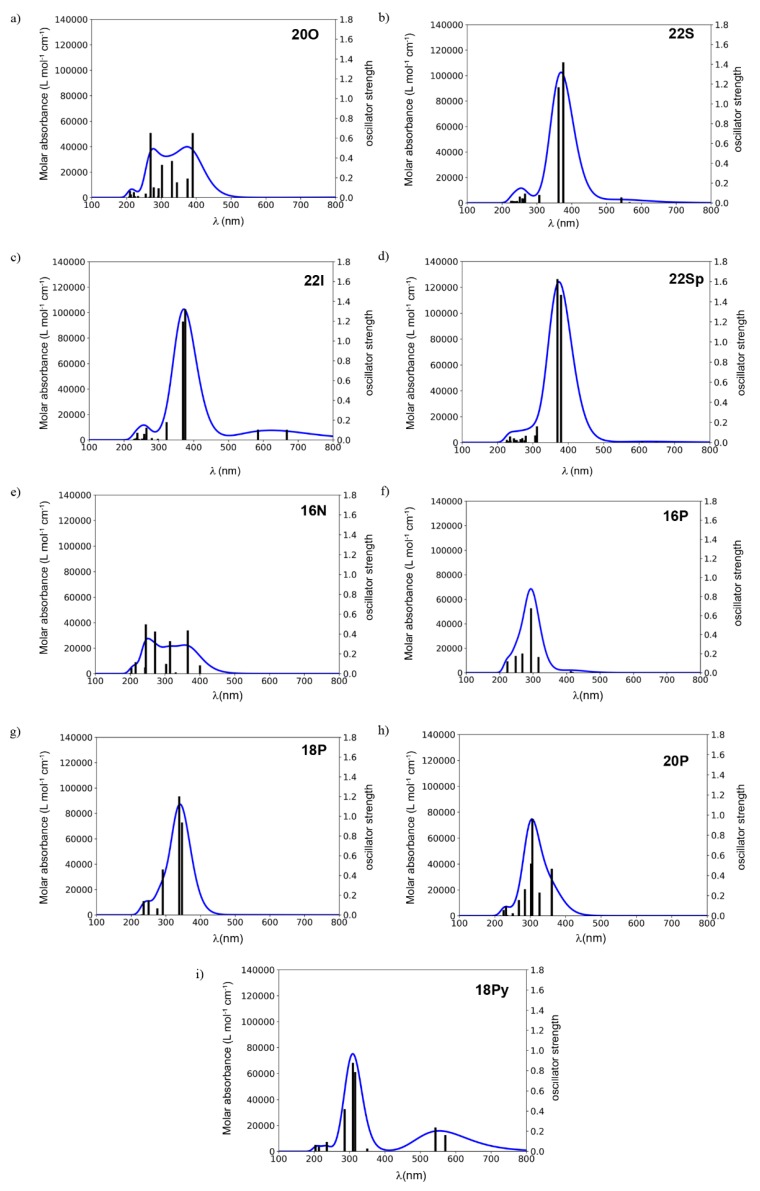
Theoretical UV/vis absorption spectra of unsubstituted Hückel porphyrinoids together with the TDDFT/CAM-B3LYP oscillator strengths: (**a**) (20)orangarin, (**b**) (22)smaragdyrin, (**c**) (22)isosmaragdyrin, (**d**) (22)sapphyrin, (**e**) (16)norcorrole, (**f**) (16)porphyrin, (**g**) (18)porphyrin, (**h**) (20)porphyrin and (**i**) porphycene.

**Figure 7:**
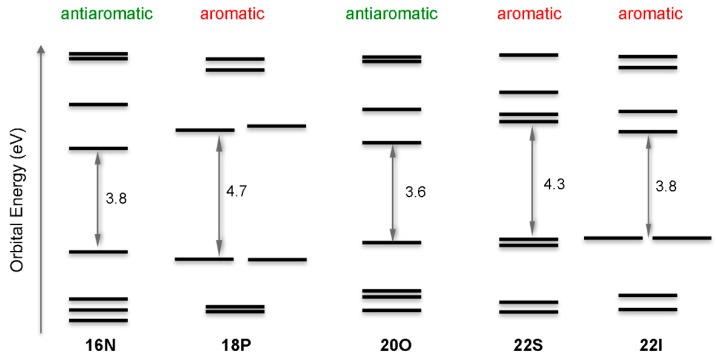
Schematic diagrams for the energy levels of selected molecular orbitals of aromatic and antiaromatic porphyrinoids. The HOMO-LUMO gap (in eV) is also shown [59].

As can be inferred from Figure 6 and Table 2, Table 3, Table 4, Table 5 and Table 6, the shape and the composition of B and Q bands differ significantly in aromatic and antiaromatic systems. In aromatic systems, B and Q bands are split. These splitting results from the quasi-degeneracy of H, H−1 and L, L+1 orbitals since the B and Q bands correspond to transitions involving those four orbitals (Figure 7). The splitting of B and Q bands in *x*,*y* component is also reflected in the polarization under which the associated electronic transitions are allowed. As shown in Table 3, the fourth electronic transition of **18P** is associated with the *y* component of the B band. The symmetry of the initial and final orbitals involved in this electronic transition indicates that the fourth transition is allowed under *y*-polarization. In addition, the large magnitude of *y*-component of the electronic transition dipole moment allows for high intensity. A similar analysis for the third electronic transition at 347 nm yields an intense *x*-polarized B band. In **18P**, **22S** and **22I**, the three main absorption bands correspond to the B bands and one high-energy electronic transition. The most important high-energy electronic transitions in **22I** and **22S** correspond to transitions between H−4→L+4, H−4→L and H→L+3, H−1→L+6, respectively. By contrast, the most intense high-energy electronic transition in **18P** involves the H−5→L transition.

On the other hand, B and Q are not split and they are significantly weaker in antiaromatic species than in aromatic systems. This phenomenon is a consequence of the low symmetry of the molecule and the non-degeneracy of H, H−1 and L, L+1 orbitals, respectively (Figure 7) [60]. As indicated by their non-zero associated oscillator strength, the Q band of **20O** and **16N** is not fully forbidden. The most intense absorption bands correspond to high-energy electronic transitions in the B band. In Table 3, the fourth electronic transition of **16N** (B band, *f*_osc_ = 0.436) is only slightly weaker than the tenth electronic transition (high-energy electronic transition, *f*_osc_ = 0.496). In **16N**, the three most intense absorption bands are associated to the following electronic transitions involving large differences in orbital sequence: H→L+4 > H→L+2 > H−4→L. In the case of **20O**, the B band and 12th electronic transition have nearly the same intensity (*f*_osc_ = 0.65), as can be seen in Table 5 and Figure 6. However, the B band of **20O** is associated to H−1→L+1 transition under *z*-polarization, while the 12th electronic transition corresponds to a H→L+2 transition, which is allowed under *y* polarization. The third main absorption band in **20O** is associated to H−2→L transition.

**Figure 8:**
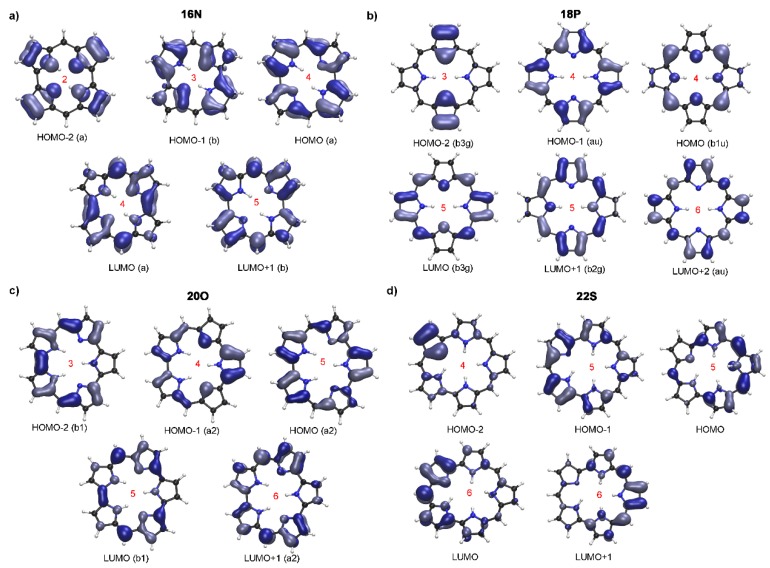
Molecular orbitals for aromatic and antiaromatic Hückel porphyrinoids: (**a**) (16)norcorrole, (**b**) (18)porphyrin, (**c**) (20)orangarin, (**d**) (22)smaragdyrin. The values in red indicate the number of nodal planes in each orbital.

To gain more insight in the intensity of electronic transitions and the nature of the involved orbitals, we applied the perimeter model [61]. The perimeter or free-electron model is an extension of the one-dimensional particle in a box problem to a particle in a ring model. The electrons are confined to a ring of constant negative potential with zero potential outside [62]. The number of nodal planes *k* of an orbital is half of the number of nodes *q* of the wave function, which in turn determines the absolute value of the angular momentum. Within the frame of the perimeter model, the number of nodal planes of the frontier orbitals is employed to determine whether a transition is allowed or not. If the difference in number of nodal planes between the initial and final orbitals equals 0 or ±1, then the transition is allowed. Otherwise, the transition is predicted to be forbidden (i.e., Δ*k >* 1) in case of absorption. Despite its very elegant formulation, the perimeter model suffers from several shortcomings that mostly impact antiaromatic systems and to a lesser extent aromatic systems. As in aromatic systems, the most important electronic transitions (B bands) involve H−1, H and L, L+1 orbitals, Δ*k* values 0 or 1 show up, leading to allowed transitions in the model in accordance with the symmetry analysis. Finer details for aromatics cannot be completely reproduced by the perimeter model. As can be seen in Figure 8, H−1 and H orbitals of **22S** exhibit five nodal planes, whereas L and L+1 present six nodal planes. The transition H−2 to L is forbidden according to the FMO model because Δ*k* equals 2. Nevertheless, it appears with a non-zero, albeit small intensity (*f*_osc_ = 0.08). In Table 4, we observe that the electronic transition H→L+2 (*f*_osc_ = 0.65, Δ*k* = 3) in **20O** is forbidden according the perimeter model but allowed according to symmetry analysis. In antiaromatic systems, high-energy electronic transitions are important and those do not fulfill the criterion Δ*k* = 0, ±1 of the perimeter model. As a conclusion, the FMO model is too approximated for our porphyrinoid systems, although it works significantly better for explaining allowed transitions in Hückel aromatics than for Hückel antiaromatic systems.

**Figure 9:**
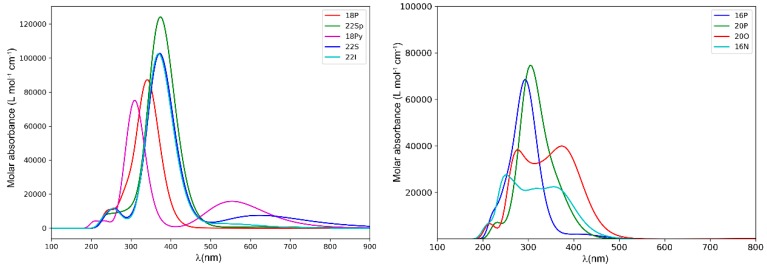
Absorption spectra of unsubstituted aromatic (**left**) and antiaromatic (**right**) Hückel porphyrinoids simulated using Gaussian functions with a halfwidth of 2686 cm^−1^.

To generalize these observations, the UV-vis spectra of **16P**, **20P**, **18Py** and **22Sp** were also computed and carefully analyzed. The characteristic properties of the main electronic transitions of these porphyrinoids are collected in Tables S7–S10. Figure 9 shows the overlay of the absorption spectra for aromatic and antiaromatic Hückel porphyrinoids. For the aromatic **22Sp**, a very intense B band is observed corresponding to electronic transitions involving H, H−1, L and L+1 orbitals. As compared to the pentapyrrolic macrocycles, the B band of aromatic (**18**)**Py** is blue shifted and lower in intensity, but similarly it involves the electronic transitions between the quasi degenerate H and H−1 to L/L+1 orbitals. In addition, the B and Q bands in both aromatic systems are split in *x*,*y* components. Remarkably, the Q band of the **18Py** is very intense among aromatic systems.

**Figure 10:**
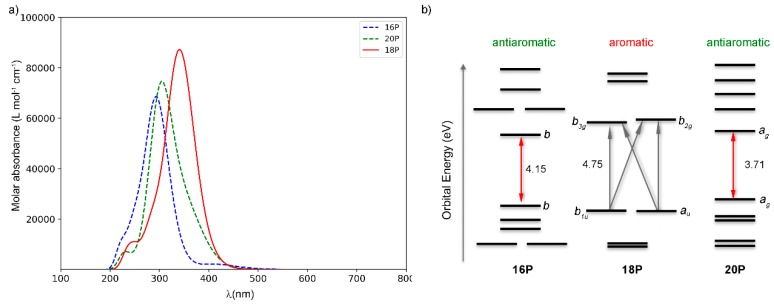
UV/vis absorption spectra of regular porphyrin (**18P**), oxidized porphyrin (**16P**) and reduced porphyrin (**20P**) (**a**) together with the MO energy diagram of selected molecular orbitals (**b**).

To assess the effect of the aromaticity on the photophysical spectra, we additionally investigate the changes in the UV-vis spectra of the archetypal (18)porphyrin upon oxidation and reduction of the macrocycle. The (16)porphyrin and (20)porphyrin are antiaromatic according to the Hückel rule and aromaticity descriptors, in contrast to the parent macrocycle which is clearly aromatic. The overlay of the absorption spectra of **18P**, **16P** and **20P** together with the MO energy diagram is shown in Figure 10. From the spectra, it is clear that **16P** and **20P** exhibit a unique blue-shifted Soret-like band at around 300 nm and no Q band. These observations are in good agreement with the experimental spectra for 16 π-electron porphyrins [63]. For **16P** and **20P**, the electronic transition between HOMO and LUMO is symmetry forbidden by the selection rule and the perimeter model with an oscillator strength of 0.00 (Tables S9 and S10). By contrast, the HOMO-LUMO transition in **18P** is allowed by symmetry and the perimeter model (Table S3). As a consequence, the most intense absorption band correspond to a H−8→L transition in **16P** and H−2→L and H→L+3 transitions in **20P**. From the schematic MO diagram, it can be inferred that the quasi-degeneracy of the frontier MOs in **18P** is broken in the antiaromatic counterparts. These findings are in line with the photophysical features ascribed to antiaromatic porphyrinoids. However, the intensity of the B-band in **16P** and **20P** is exceptionally high as compared with the remaining antiaromatic systems (**20O** and **16N**) (Figure 9).

### 2.3. Polarizability and Nonlinear Optical Properties in Hückel Porphyrinoids

At the molecular level, the quantities of interest are the polarizability (*α*) as well as the first (*β*) and second hyperpolarizability (*γ*), associated with second- and third-order nonlinear optical effects, respectively. Table 7 contains the average polarizability ($\bar{\alpha }$) and anisotropy (Δ*α*) in both static and dynamic regime at different frequencies. As can be seen, the dynamic $\bar{\alpha }$ and Δ*α* values hardly depend on the incident wavelength in the selected frequency range.

**Figure 11:**
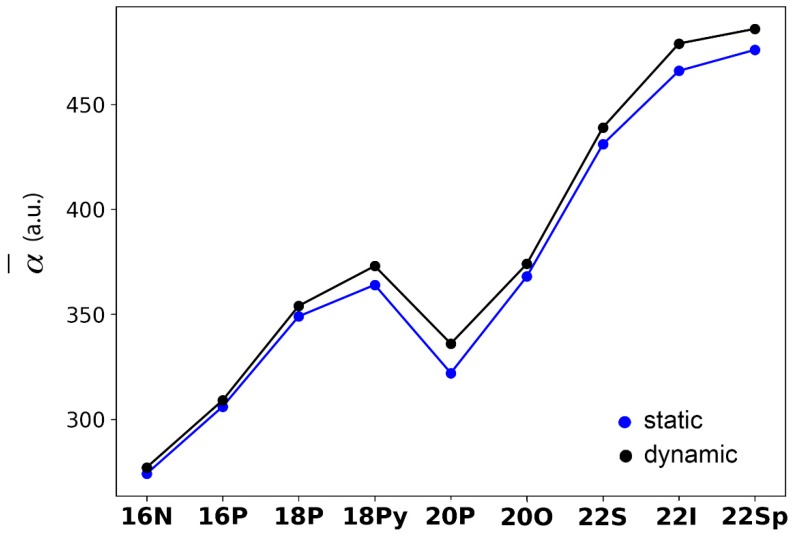
Evolution of the average polarizability in static and dynamic regime (1900 nm), both computed in gas-phase.

From Table 7, it is clear that polarizability increases with the macrocycle size, so the polarizability of pentapyrrolic macrocycles is larger than that of tetrapyrrolic compounds. Regarding the average polarizability (Figure 11), the following sequence is found: **22Sp** > **22I** > **22S** > **20O** > **18Py** > **18P** > **20P** > **16P** > **16N**. Therefore, beside the effect of the macrocycle size, polarizability increases with aromaticity, in such a way that aromatic systems are characterized by larger average polarizability and anisotropy in both static and dynamic regime than antiaromatic homologues with a similar ring size. In all cases, solvation significantly enhances both the isotropic and anisotropic polarizability.

Regarding first hyperpolarizabilities, we focus on two measurable second-order NLO responses: the hyper-Rayleigh scattering (HRS) response (*β*_HRS_) and the electric-field-induced second harmonic generation EFISHG response (*β*_//_) [64,65]. We also computed the depolarization ratio (DR), since it provides information on the geometry of the chromophore, i.e., the part of the molecule responsible for the NLO response [66]. These first hyperpolarizability characteristics both in static and dynamic regime for the different Hückel porphyrinoids are collected in Table 8.

**Figure 12:**
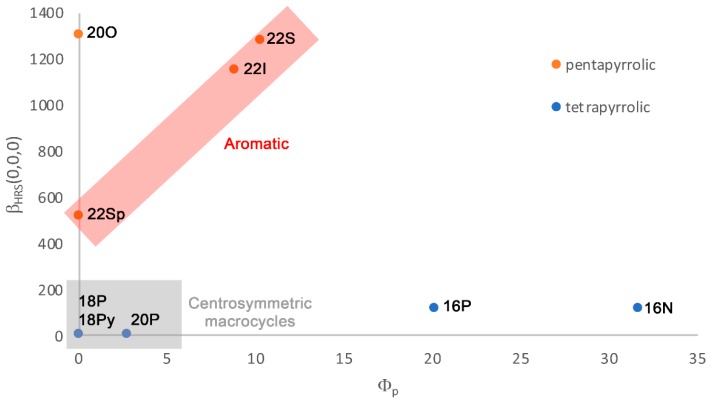
Dependence of the static *β*_HRS_ values (in a.u.) with the macrocycle size and the planarity of the macrocycle, as denoted by the torsional strain (Φ_p_ in °). For centrosymmetric structures, *β*_HRS_ equals 0.

From Table 8, it is clear that *β*_HRS_ depends on the molecular symmetry, while the relationship between *β*_HRS_ and aromaticity of the π-conjugation system is not unambiguous. For centrosymmetric structures, such as **18P**, **18Py** and **20P**, *β*_HRS_, *β*_//_ and *β*__|__ values equal zero. For non-centrosymmetric structures, the length of the conjugation pathway influences the magnitude of the first hyperpolarizability, in such a way that pentapyrrolic macrocycles exhibit larger *β*_HRS_ values than tetraphyrins. For non-centrosymmetric systems with a similar ring size and aromaticity, such as **22S**, **22I** and **22Sp** the factor determining the first hyperpolarizability is the planarity of the ring system (Figure 12). The totally planar **22Sp** (Φ_P_ = 0°) exhibit smaller ∣*β*_HRS_∣, ∣*β*_//_∣ and ∣*β*__|__∣ values than **22I** (Φ_P_ = 8.8°), which in turns show smaller first hyperpolarizability values than **22S** (Φ_P_ = 10.3°). As such, besides the molecular symmetry and the conjugation pathway length, non-planar structures exhibit enhanced first hyperpolarizabilities. It is important to note that the exalted magnitude of *β*_HRS_, *β*_//_, *γ*__|__, *γ*_//_ and *γ*__|__ of **20O** at 1900 nm indicates that the system is close to resonance. The first resonance equals the half of the first excitation energy. In our selected systems, the first excitation energy is associated with the Q band (**16N**: 1.18 eV, **20O**: 1.29 eV, **20P**: 1.33 eV, **16P**: 1.63 eV, **22I**: 1.86 eV, **22Sp**:1.91 eV, **18Py**: 2.17 eV, **22S**: 2.19, **18P**: 2.22 eV). Therefore, the first resonance energy of **16N** (0.59 eV), **20O** (0.65 eV) and **20P** (0.67 eV) are significantly smaller than those of **18P** (1.11 eV), **22S** (1.10 eV) and **22I** (0.93 eV). Though the first resonance energy of both **16N** and **20O** is close to the energy of the incident light (0.654 eV), resonance is only observed for **20O** because the oscillator strength of the first electronic transition of **16N** and **20P** is zero, whereas it is non-zero for **20O** (*f_osc_* = 0.007). Similar to the polarizability, solvation enhances the magnitude of first and second hyperpolarizability.

**Figure 13:**
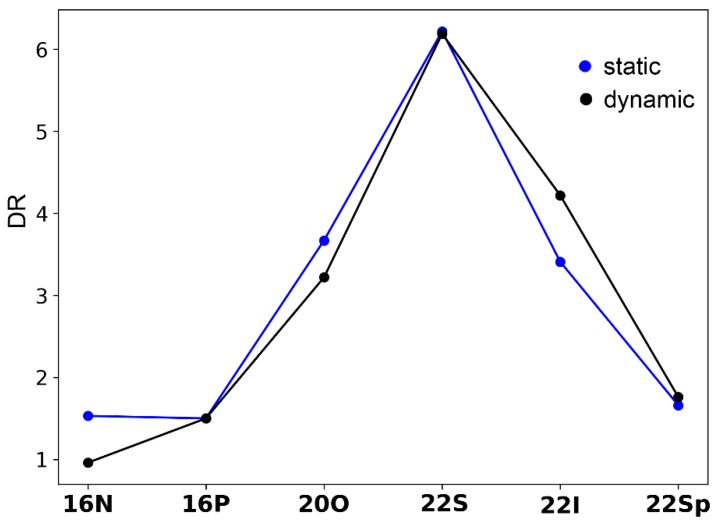
Evolution of the depolarization ratio (DR) in static and dynamic regime in solvent (2128 nm).

The effect of aromaticity on the depolarization ratio (DR) seems to be more straightforward, in such a way that aromatic porphyrinoids exhibit larger DR values than antiaromatic ones for non-centrosymmetric structures. In the off-resonance region (e.g., 2128 nm, Figure 13) the DR value decreases in the following order: **22S** > **22I** > **20O** > **22Sp** > **16P** > **16N**. From this order, it seems that pentapyrrolic macrocycles exhibit larger DR than tetrapyrrolic systems. In contrast to *β*_HRS_, the depolarization ratio does not vary significantly upon solvation.

**Figure 14:**
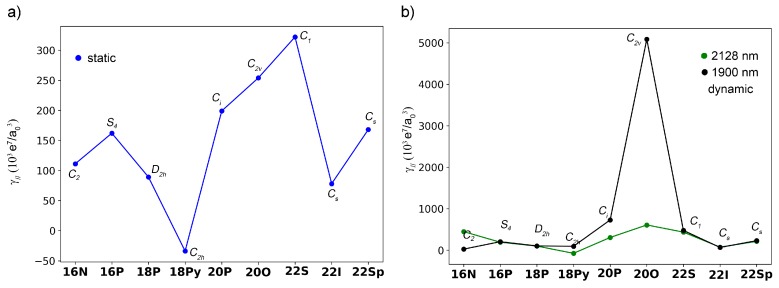
Evolution of the longitudinal component of the second hyperpolarizability (*γ*_//_) in static (**a**) and dynamic regime (1900 nm and 2128 nm) (**b**). The symmetry point group for each system is also shown.

Regarding the second hyperpolarizability, we observe two different trends in the static and in dynamic regime, as shown in Figure 14. For instance, **22S** is characterized by the largest *γ*_//_ value in the static regime, followed by **20O** and **20P**. However, in the dynamic regime, the following decreasing order for *γ*_//_ is found: **20O** > **20P** > **22S** > **22Sp** > **16P** > **18P** > **22I** > **16N** > **18Py**. Here, it should be again noted that **20O** is close to resonance at 1900 nm. In contrast to the rest of porphyrinoids, **18Py** exhibits a negative value for *γ*_//_ in both static and dynamic regime. From these results, it is difficult to assess the role of aromaticity, π-conjugation length and symmetry on the second hyperpolarizability *γ*_//_. Even in the static regime, it does not seem to be a clear structure-property relationship for *γ*_//_. Nevertheless, it is clear that solvation and the wavelength of the incident light largely influences the value of the second hyperpolarizability.

From the frequency-dispersion plots (Figures S3–S8) and the NLO properties computed at different wavelengths (Tables S10 and S11), it is clear that *β*_HRS_ and DR values are more dependent on the wavelength of the incident light than properties associated to the polarizability. Moreover, the properties associated to the first and second hyperpolarizability are more frequency-dependent than the DR. As shown in Table 8, we observe exalted *β*_HRS_, *β*_//_ and *γ*_//_ for **20O**, whereas this enhancement is associated to a slight variation in DR value. Given the energy of the first resonance in **16N**, **16P**, **18P**, **18Py**, **20P**, **20O**, **22S**, **22Sp** and **22I**, we can assume that all the systems are far from resonance between 0 and 0.413 eV. For the region between 0.583 eV (2128 nm) and 0.653 eV (1900 nm), the frequency dispersion of the first and second hyperpolarizability of antiaromatic systems (**16N**, **20P** and **20O**) is found to be more important than in aromatic ones (**18P**, **18Py**, **22Sp**, **22I** and **22S**). As mentioned above, this phenomenon results from the similitude between the energy of the first resonance of **16N** and **20O** and the energy of the incident light at 1900 nm, respectively. Hence, these second-order properties can be tuned by varying the range of the incident wavelength.

Summarizing, for the selected set of Hückel porphyrinoids, aromatic porphyrinoids are found to display larger $\bar{\alpha }$ and Δ*α* than antiaromatic ones with a similar ring size. However, *β*_HRS_, *β*_//_ and *γ*_//_ depend on three main molecular factors (molecular symmetry, macrocycle size and planarity) in addition to the incident wavelength and solvation, what makes difficult to establish a clear rationalization of NLO properties in terms of (anti)aromaticity.

### 2.4. Substituent Effect on the Structure-Property Relationships

As *meso*-substitution can lead to significant change in properties of porphyrinoids [3,41], we have investigated the evolution of the aromaticity, absorption spectrum and nonlinear optical properties of *meso*-pentafluorophenyl-substituted **16N**, **18P**, **20O**, **22S** and **22I**. These aryl substituents are commonly used in the synthesis of expanded porphyrins [3]. In this section, it is important to note that our goal is to assess if the devised structure-property relationships hold for real-world *meso*-substituted porphyrinoids. A detailed investigation of the effect of peripheral substituents on the NLO properties is out of the scope of the present article, although it is expected that the presence of strong electron-withdrawing and -releasing groups located on opposite sides of the porphyrinoid skeleton will led to a dramatic increase of the NLO responses [22]. Here, only a subset of porphyrinoids is considered, namely **16N**, **18P**, **20O**, **22I** and **22S**.

**Figure 15:**
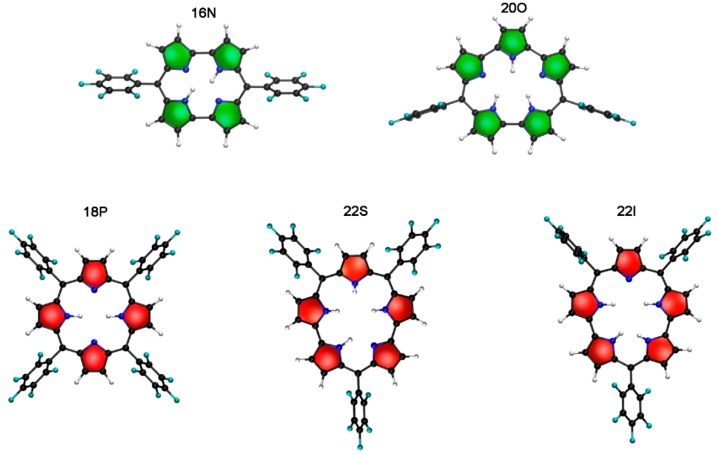
Hückel conformations computed for the selected *meso*-substituted porphyrinoids. Red five-membered rings are linked to aromatic structures, whereas green ones to antiaromatic configurations.

The optimized geometries of *meso*-substituted porphyrinoids are shown in Figure 15 and the aromaticity descriptors are collected in Table 9. Aside **16N**, which becomes more planar, the planarity of the remaining Hückel systems decreases upon *meso*-substitution. Despite a closure of HOMO-LUMO gap, we observe the same trends as for the unsubstituted systems: **18P**, **22S** and **22I** are aromatic (diatropic) whereas **16N** and **20O** are antiaromatic (paratropic). Therefore, the (anti)aromaticity of the macrocycles hardly changes upon attachment of the pentafluorophenyl groups.

**Figure 16:**
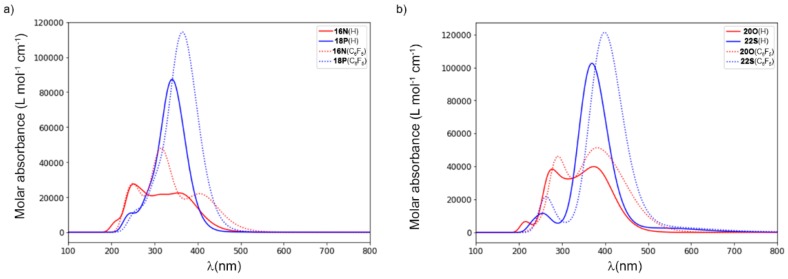
Evolution of the UV/vis absorption spectra upon *meso*-substitution for (**a**) (16)norcorrole and (18)porphyrin; (**b**) [20]orangarin and [22]smaragdyrin.

Regarding the absorption spectra, we observe that the intensity of the absorption bands of **16N**, **18P**, **20O**, **22S** and **22I** are enhanced upon *meso*-substitution, irrespective of the aromaticity or antiaromaticity (Figure 16). In addition, the absorption bands are red-shifted, indicating that the excitation energies are slightly reduced. For instance, the energy associated to the first electronic transition of **16N** and **22S** change from 1.18 to 1.16 eV and from 2.19 to 2.07 eV, respectively, upon substitution. Nevertheless, the absorption spectra of *meso*-substituted macrocycles share the same features as those of unsubstituted porphyrinoids and the fingerprints of (anti)aromaticity on the photophysical properties are very similar.

Table 10 reports the non-linear optical properties (*β*_HRS_, DR, *β*_//_ and *γ*_//_) of *meso*-substituted **16N**, **18P**, **20O**, **22S** and **22I** in gas and solvent-phase. In the static and dynamic regime, we can classify the *meso*-substituted macrocycles according to decreasing *β*_HRS_ and *β*_//_ as **22I** > **22S** > **20O** > **16N** > **18P**. This change in trends can be related to the change of symmetry induced by *meso*-substitution. The *meso*-substitution increases the asymmetry of the systems especially for the macrocycles with an odd number of substituents (**22S** and **22I**). In addition, due to the presence of *meso*-aryl groups, excitation energies of the porphyrinoids are shifted. As a result, the energy of the first resonance of *meso*-substituted **16N** and **20O** thus do not correspond exactly to 0.653 eV. As in unsubstituted porphyrinoids, solvation enhances the magnitude of *β*_HRS_ and *β*_//_. Interestingly, we found distinct tendencies for DR values of *meso*-substituted porphyrinoids in the static and in the dynamic regime. In the dynamic regime, the DR is correlated with the aromaticity of the system, while the DR of **20O** and **16N** exceed the DR of **22S** and **18P** in the static regime, respectively. In addition, aromatic *meso*-substituted systems are characterized by larger *γ*_//_ values in the dynamic regime. However, in the static regime, **16N** exhibits larger *γ*_//_ values than **18P**. This might be related to the predominance of the lack of symmetry in **16N** to the aromaticity of **18P**. However, according to *γ*_//_ in the static regime, we found **22S** > **22I** > **20O** > **16N** > **18P**. This order suggests that a lack of symmetry is the key factor to obtain large *γ*_//_ in combination with aromaticity. Nevertheless, in case of a large difference in the molecular symmetry, the lack of symmetry is the predominant factor.

## 3. Computational Details

All the calculations were carried out with Gaussian 09.D01 software package using the M06 [67] or CAM-B3LYP [68] functionals together with the split-valence basis sets [6-31G(d,p) and 6-311+G(d,p)] [69]. Geometry optimization and vibrational frequency calculations were performed at the M06/6-31G(d,p) level of theory. All the structures correspond to minima on the potential energy surface with no imaginary frequencies. The performance of the M06 hybrid functional on the geometries and relative conformational energies of expanded porphyrins was assessed in our previous benchmarks from comparison with experiment [40,41].

The harmonic oscillator model of aromaticity (HOMA) was evaluated as a structural descriptor of aromaticity [31]:

(1) $$\mathrm{HOMA}=1-\frac{\alpha }{n}\sum_{i=1}^{n}{\left(R_{opt}-R_{i}\right)}^{2}$$

where *α* is an empirical constant fixed for each type of bond and *n* corresponds to the number of bonds taken into account in the summation. *R_i_* denotes the running bond length along the annulene-type conjugation pathway. HOMA equals 0 for non-aromatic systems, whereas HOMA = 1 for fully aromatic ones with all bonds equal to the optimal value *R_opt_*. For C-C bonds, *α* = 257.7 and *R_opt_* = 1.388 Å, whereas *α* = 93.52 and *R_opt_* = 1.334 Å for C-N bonds [70].

**Scheme 1:**
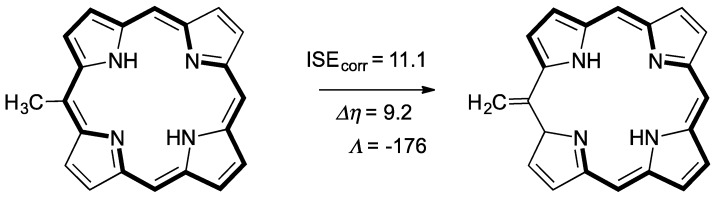
Reaction used to evaluate several aromaticity descriptors in octaphyrins. ISE_corr_ and Δ*η* are given in kcal mol^−1^ and *Λ* in ppm cgs.

The isomerization method was applied to compute the isomerization stabilization energies (ISE) [55], magnetic susceptibility exaltation (*Λ*) [71] and relative hardness (Δ*η*) [72] of unsubstituted species (Scheme 1). This method is based on the difference in properties between a methyl derivative of the conjugated system and its nonaromatic exocyclic methylene isomer. For the energetic descriptor, *syn*-*anti* corrections are required due to the *cis-trans* diene mismatches in the methyl and methylene isomer [55]. The *syn*-*anti* corrections were calculated as the energy difference between the dihydrogen derivative of *meso*-methyl species and its respective nonaromatic isomer. To obtain the susceptibility exaltation (*Λ*), the magnetic susceptibilities were evaluated using the CGST method [73] at the CAM-B3LYP/6-311+G(d,p) level of theory. For the evaluation of relative hardness, HOMO and LUMO energies were employed to evaluate the hardness of the methyl and methylene derivatives in the frame of the isomerization scheme.

Nucleus-independent chemical shift (NICS) values [32,74] were computed at the geometrical center of the heavy atoms along the annulene pathway of the macrocycle and at 1 Å above and below the ring center, together with its out-of-plane tensor component, by employing the GIAO method [75] together with the CAM-B3LYP functional together with the 6-311+G(d,p) basis set. The anisotropy of the induced current density (AICD) method was applied to provide a complementary view of the induced delocalization of π-electrons [56,57]. For the NICS and ACID calculations, the external magnetic field is applied perpendicular to the molecular plane.

As additional geometrical descriptors, we computed the average dihedral angle between neighboring pyrrole rings (Φ_p_) as a measure of the torsional ring strain [37] and the π-conjugation index (*Π*) to quantify the extent of the effective overlap of neighboring p orbitals [1]. *Π* equals 1 for a completely planar system, it is positive for any Hückel (double-sided) conformation and negative for any Möbius (single-sided) surface. Normally, macrocyclic aromaticity is associated with porphyrinoids having *Π* values higher than 0.3.

Excitation energies and corresponding oscillator strengths (*f*_osc_) of main electronic transitions were computed using time-dependent DFT (TD-DFT) [76] at the CAM-B3LYP/6-311+G(d) level of theory. Polarizabilities and hyperpolarizabilities were evaluated with the time-dependent DFT (TDDFT) and the coupled-perturbed Kohn-Sham (CPKS) methods to obtain their dynamic and static NLO responses, respectively [66]. Several benchmark studies pointed out that the long-range corrected CAM-B3LYP functional outperforms other exchange-correlation functionals in the evaluation of NLO properties of organic molecules, including porphyrinoid compounds [21,23,77].

The molecular properties known as the electric dipole polarizability (*α*), first (*β*) and second (*γ*) hyperpolarizabilities, are defined by an equation describing the change in the components of the electric dipole moment Δ*μ_ζ_* (*ζ* = x,y,z), which results from the application of external electric fields **F** (Equation (2)):

(2) $$\Delta \mu_{\zeta }=\overset{x,y,z}{\underset{\eta }{\sum }}\alpha_{\zeta \eta }\left(-\omega_{\sigma };\omega_{1}\right)F_{\eta }\left(\omega_{1}\right)+\frac{1}{2!}\overset{x,y,z}{\underset{\eta \chi }{\sum }}\beta_{\zeta \eta \chi }\left(-\omega_{\sigma };\omega_{1},\omega_{2}\right)\;F_{\eta }\left(\omega_{1}\right)F_{\chi }\left(\omega_{2}\right)+\frac{1}{3!}\overset{x,y,z}{\underset{\eta \chi \xi }{\sum }}\gamma_{\zeta \eta \chi \xi }\left(-\omega_{\sigma };\omega_{1},\;\omega_{2},\;\omega_{3}\right)F_{\eta }\left(\omega_{1}\right)F_{\chi }\left(\omega_{2}\right)F_{\xi }\left(\omega_{3}\right)+\ldots$$

where F*_η_*(*ω*_1_) is the amplitude of the field oscillating at pulsation *ω*_1_ and applied in the *η* direction and *ω_σ_* = Σ_i_*ω*_i_.

The quintessence of the polarizability tensor *α*_ij_ is represented by its invariants: the average polarizability $\bar{\alpha }$, defined as (Equation (3)):

(3) $$\bar{\alpha }=\frac{1}{3}\underset{i=x,y,z}{\sum }\alpha_{ii}$$

and its anisotropy Δ*α* defined through the relation (Equation (4)):

(4) $$\Delta \alpha^{2}=\frac{1}{2}\left[{\left(\alpha_{xx}-\alpha_{yy}\right)}^{2}+{\left(\alpha_{xx}-\alpha_{zz}\right)}^{2}+{\left(\alpha_{zz}-\alpha_{yy}\right)}^{2}+6\alpha_{xz}^{2}+6\alpha_{yz}^{2}+6\alpha_{xy}^{2}\right]$$

The real part of *α*(−*ω*,*ω*) is related to the refraction.

First and second hyperpolarizabilities can be split in a longitudinal (//) and transversal (_|_) component. The longitudinal component of *β* is related to the second harmonic generation (SHG) and the electric-field induced SHG (EFISHG). The EFISHG measurement gives insight in the projection of the vector part of *β* on the dipole moment vector *μ* (Equation (5))*.*

(5) $$\beta_{\|}\left(-2\omega ;\omega ,\omega \right)=\frac{1}{5}\overset{x,y,z}{\underset{i}{\sum }}\frac{\mu_{i}}{\left\|\mu \right\|}\overset{x,y,z}{\underset{j}{\sum }}\left(\beta_{ijj}+\beta_{jij}+\beta_{jji}\right)=\frac{3}{5}\overset{x,y,z}{\underset{i}{\sum }}\frac{\mu_{i}\beta_{i}}{\left\|\mu \right\|}$$

where ‖μ‖ is the norm of the dipole moment and $\mu_{i}$ and $\beta_{i}$ the components of the μ vector and β vector part, respectively. The transversal component is given by Equation (6):

(6) $$\beta_{\_|\_}\left(-2\omega ;\omega ,\omega \right)=\frac{1}{5}\underset{i,j=x,y,z}{\sum }\frac{\mu_{i}}{\left\|\mu \right\|}\left(2\beta_{jji}-\beta_{jij}\right)$$

In analogy, we considered the *γ*_//_ and *γ*__|__ quantities defined as (Equations (7) and (8)):

(7) $$\gamma_{\|}\left(-2\omega ;\omega ,\omega ,0\right)=\frac{1}{15}\underset{i,j=x,y,z}{\sum }(\gamma_{iijj}+\gamma_{ijij}+\gamma_{ijji})$$

(8) $$\gamma_{⊥}\left(-2\omega ;\omega ,\omega ,0\right)=\frac{1}{15}\underset{i,j=x,y,z}{\sum }(2\gamma_{ijji}-\gamma_{iijj})$$

As *β*_//_, the longitudinal component of the second hyperpolarizability *γ*_//_ is also associated to EFISHG. By contrast, the first hyperpolarizability related to Hyper-Raleigh scattering (*β*_HRS_) and the depolarization ratio (DR) are both defined for neutral and charged molecules. *β*_HRS_ corresponds to the orientational average of the first hyperpolarizability over all molecular orientations (Equation (9)). Experimentally, *β*_HRS_ corresponds to the second-order NLO response that can be extracted from Hyper-Raleigh scattering, namely the intensity of the incoherent scattering at frequency 2*ω* on incidence of a laser with frequency *ω*. DR (Equation (10)) provides insight over the geometry of the part of the molecule responsible for the NLO response [64].

(9) $$\beta_{HRS}\left(-2\omega ;\omega ,\omega \right)=\sqrt{\langle \beta_{ZZZ}^{2}\rangle +\langle \beta_{ZXX}^{2}\rangle }$$

(10) $$DR=\frac{\langle \beta_{ZZZ}^{2}\rangle }{\langle \beta_{ZXX}^{2}\rangle }$$

where $\langle \beta_{\mathrm{ZZZ}}^{2}\rangle$ and $\langle \beta_{\mathrm{XZZ}}^{2}\rangle$ correspond to the orientational averages of the *β*. 

## 4. Conclusions

In this work, we have investigated the aromaticity, photophysical properties and nonlinear optical properties of a series of porphyrinoids (**16N**, **16P**, **18P**, **18Py**, **20P**, **20O**, **22S**, **22I** and **22Sp**) differing in conjugation pathway length and aromaticity but sharing a Hückel topology. To explore the link between aromaticity, photophysical properties and NLO properties, the aromaticity of each porphyrinoid has been first assessed using an exhaustive set of aromaticity descriptors. Then, the features of UV/vis absorption spectra, NLO properties and polarizability of Hückel porphyrinoids have been rationalized in terms of aromaticity patterns.

Due to the multidimensional character of aromaticity, we used several aromaticity descriptors based on the energetic, magnetic, structural, reactivity and electronic criteria. In good agreement with the annulene model, our aromaticity descriptors indicate that **18P**, **18Py**, **22S**, **22I** and **22Sp** are fully aromatic, whereas **16N**, **20O** and the oxidized and reduced form of porphyrin (**16P** and **20P**) are antiaromatic. We observed some drawbacks for the isomerization stabilization energies since they are very sensitive to structural distortions and cannot distinguish properly between aromatic and antiaromatic systems.

Regarding photophysical properties, aromatic systems are characterized by UV/vis spectra with Q bands and very sharp and intense B bands, mainly due to transitions involving the H, H−1 and L, L+1 molecular orbitals. By contrast, antiaromatic systems display significantly weaker absorption bands associated to high-energy electronic transitions, whereas the B band is significantly weaker and broader than in the absorption spectrum of aromatic systems. Only for the antiaromatic **16P** and **20P**, the intensity of the B band is significant, but the electronic transition between HOMO and LUMO is symmetry forbidden in contrast to the aromatic **18P**. As such, a close relationship is found between the (anti)aromaticity and the absorption spectra in Hückel porphyrinoids.

In comparison to the photophysical properties, the influence of aromaticity on the nonlinear optical properties is significantly more complex. On one hand, aromatic systems are characterized by larger $\bar{\alpha }$ and Δ*α* values than antiaromatic systems with similar macrocycle size and the magnitude of these quantities are nearly invariant with the wavelength of the incident light. Due to differences in symmetry and resonance effects, we could not determine the influence of aromaticity on NLO properties. Furthermore, *β*_HRS_, *β*_//_ and *γ*_//_ are frequency-dependent and their magnitude is determined by the interplay between symmetry, macrocycle size and planarity. All the NLO properties are significantly enhanced upon solvation.

Finally, the influence of *meso*-substitution on the aromaticity, UV/VIS and NLO of the macrocycles was investigated. Despite keeping the same trends in aromaticity, we observe an enhancement and red shift of the absorption bands upon *meso*-substitution. The trends change for several NLO properties since some substituted systems are not in resonance anymore and the symmetry of the systems changed upon attachment of aryl substituents. Nevertheless, among the investigated Hückel porphyrinoids, aromatic *meso*-substituted systems are characterized by larger DR, *β*_HRS_, *β*_//_ and *γ*_//_ values than antiaromatic ones.

At present, there is some evidence that larger two-photon absorption (TPA) cross sections are observed for aromatic expanded porphyrins as compared to the corresponding antiaromatic congeners (11). However, a generalization of this potentially useful relationship in a systematic way has yet to be made. The complex structure–property relationship between aromaticity and nonlinear optical properties for Hückel porphyrinoids pinpoint future research.

## Figures and Tables

**Table 1:** Energetic, reactivity, magnetic, structural and electronic properties of unsubstituted porphyrinoids *^a^*.

πe *^b^*	Δ*η*	*Λ*	NICS(0)	NICS(1)	NICS_zz_(1)	HOMA	*Π*	Φ_p_	ΔE_H-L_ *^c^*	AV1245 *^d^*	AV *_min_* *^d^*
**16N**	−3.0	78.8	23.1	18.9	62.3	0.610	0.44	31.7	3.76	1.262	0.136
**16P**	−11.9	27.5	9.4	7.6	29.2	0.634	0.74	20.2	4.15	0.732	0.294
**18P**	9.2	−175.8	−14.9	−13.7	−38.5	0.880	1.00	0.0	4.75	2.139	1.271
**18Py**	9.8	−147.0	−13.3	−12.5	−13.9	0.878	0.99	0.0	4.07	2.114	1.399
**20P**	−17.8	156.3	18.7	16.7	52.4	0.641	0.93	2.8	3.71	1.226	0.856
**20O**	−13.9	128.6	16.3	14.0	44.6	0.780	1.00	0.0	3.27	1.352	0.267
**22S**	2.2	−140.6	−9.6	−9.2	−24.6	0.892	0.88	10.3	4.25	2.066	0.794
**22Sp**	13.7	−249.6	−14.3	−13.4	−37.7	0.882	1.00	0.0	4.03	2.124	1.137
**22I**	1.9	−249.1	−13.6	−12.8	−35.7	0.901	0.91	8.8	3.85	2.435	0.963

*^a^* Δ*η* are given in kcal mol^−1^, *Λ* in ppm cgs and NICS indices in ppm. *^b^* Number of π-electrons along the classical conjugation pathway. *^c^* HOMO-LUMO energy difference (in eV) evaluated from CAM-B3LYP single-point calculations. *^d^* The electronic aromaticity indices were computed along the annulene conjugation pathway.

**Table 2:** Properties of the main electronic transitions of neutral unsubstituted (16)norcorrole (**16N**) *^a^*.

Excitation	Transition	Contribution (%)	*λ* *^b^*	*f*_osc_ *^c^*	*E* *^d^*	Polarization	Assignment
**1**	H→L	98.69	1052.37	0.000	1.18	z	Q
**2**	H−4→L−4	6.96	400.45	0.083	3.10	z	
	H−2→L+1	3.33				x,y	
	H−1→L	84.53				x,y	
**3**	H−7→L	5.70	388.11	0.000	3.19	x,y	
	H−2→L	85.09				z	
	H−1→L+1	4.69				z	
**4**	H−4→L	2.11	365.39	0.436	3.39	z	B
	H→L+2	94.22				z	
**5**	H−5→L+2	2.51	330.87	0.013	3.73	x,y	
	H−4→L	2.36				z	
	H−3→L	90.21				x,y	
**7**	H→L+4	41.38	314.52	0.327	3.38	z	
**10**	H−4→L	81.08	271.66	0.426	3.81	z	
**13**	H→L+4	81.19	242.94	0.496	4.00	z	

*^a^* Computed at the TDDFT/CAM-B3LYP level of theory using the IEFPCM scheme (solvent = CH_2_Cl_2_) on ground state geometries optimized *in vacuum*; *^b^* Absorption wavelength (*λ* in nm); *^c^* Oscillator strength (*f*_osc_); *^d^* Vertical transition energies (*E* in eV).

**Table 3:** Properties of the main electronic transitions of neutral unsubstituted (18)porphyrin (**18P**) *^a^*.

Excitation	Transition	Contribution (%)	*λ* *^b^*	*f*_osc_ *^c^*	*E* *^d^*	Polarization	Assignment
**1**	H−1→L	47.52	559.66	0.008	2.22	x	Qx
	H→L+1	52.62				x	
**2**	H−1→L+1	51.15	514.30	0.002	2.41	y	Qy
	H→L	48.60				y	
**3**	H−4→L+1	12.58	346.60	0.937	3.58	x	Bx
	H−1→L	48.22				x	
	H→L+1	39.86				x	
	L+1→H	2.46				x	
**4**	H−1→L+1	49.12	338.53	1.202	3.66	y	By
	H→L	52.30				y	
**5**	H−3→L+2	4.98	314.18	0.000	3.95	/	
	H−2→L+1	91.50				/	
	H→L+2	2.08				z	
**6**	H−5→L	80.03	291.26	0.461	4.26	y	
**16**	H−5→L	85.33	250.14	0.149	4.96	y	
**18**	H−4→L	96.34	235.80	0.140	5.26	x	

*^a^* Computed at the TDDFT/CAM-B3LYP level of theory using the IEFPCM scheme (solvent = CH_2_Cl_2_) on ground state geometries optimized *in vacuum*; *^b^* Absorption wavelength (*λ* in nm); *^c^* Oscillator strength (*f*_osc_); *^d^* Vertical transition energies (*E* in eV).

**Table 4:** Properties of the main electronic transitions of neutral unsubstituted (20)orangarin (**20O**) *^a^*.

Excitation	Transition	Contribution (%)	*λ* *^b^*	*f*_osc_ *^c^*	*E* *^d^*	Polarization	Assignment
**1**	H→L	97.55	954.1	0.007	1.29	y	Q
**2**	H−1→L+1	98.45	389.6	0.652	3.18	z	B
**3**	H−1→L	6.86	375.1	0.192	3.31	y	
	H−4→L	83.88				y	
**4**	H−3→L	42.60	344.5	0.154	3.60	z	
	H−2→L	28.92				z	
**5**	H−3→L	27.26	330.3	0.369	3.75	z	
	H−2→L	66.51				z	
**6**	H−4→L	67.52	301.6	0.329	4.11	y	
**12**	H→L+2	93.84	269.0	0.653	4.61	y	

*^a^* Computed at the TDDFT/CAM-B3LYP level of theory using the IEFPCM scheme (solvent = CH_2_Cl_2_) on ground state geometries optimized *in vacuum*; *^b^* Absorption wavelength (*λ* in nm); *^c^* Oscillator strength (*f*_osc_); *^d^* Vertical transition energies (*E* in eV).

**Table 5:** Properties of the main electronic transitions of neutral unsubstituted (22)smaragdyrin (**22S**) *^a^*.

Excitation	Transition	Contribution (%)	*λ* *^b^*	*f*_osc_ *^c^*	*E* *^d^*	Polarization	Assignment
**1**	H−1→L	52.81	566.44	0.008	2.19	y	Qy
	H→L+1	42.44					
**2**	H−1→L+1	27.12	542.5	0.056	2.29	x	Qx
	H→L	68.23					
**3**	H−1→L	44.57	375.78	1.420	3.30	y	By
	H→L+1	55.86					
**4**	H−1→L+1	69.40	362.30	1.168	3.42	x	Bx
	H→L	28.69					
**5**	H−3→L	9.47	307.14	0.081	4.04	x	
	H−2→L	77.50					
**8**	H→L+3	61.17	265.99	0.094	4.66	x	
	H−1→L+6	9.28					
**11**	H−5→L	24.76	257.92	0.046	4.81	x	
	H−2→L+1	11.93					
	H−9→L	9.68					
	H−1→L+4	15.21					

*^a^* Computed at the TDDFT/CAM-B3LYP level of theory using the IEFPCM scheme (solvent = CH_2_Cl_2_) on ground state geometries optimized *in vacuum*; *^b^* Absorption wavelength (*λ* in nm); *^c^* Oscillator strength (*f*_osc_); *^d^* Vertical transition energies (*E* in eV).

**Table 6:** Properties of the main electronic transitions of neutral unsubstituted (22)isosmaragdyrin (**22I**) *^a^*.

Excitation	Transition	Contribution (%)	*λ* *^b^*	*f*_osc_ *^c^*	*E* *^d^*	Polarization	Assignment
**1**	H−1→L+1	28.61	667.43	0.103	1.86	z	Q
	H→L	70.93				z	
**2**	H−1→L	72.54	584.78	0.105	2.12	x,y	Q
	H→L+1	26.94				x,y	
**3**	H−1→L	28.57	375.89	1.321	3.30	x,y	B
	H→L+1	73.62				x,y	
**4**	H−1→L+1	69.35	369.59	1.195	3.35	z	B
	H→L	28.97				z	
**5**	H−2→L	93.02	322.38	0.180	3.85	z	
	H−1→L+1	2.46				z	
**9**	H−4→L	29.11	261.61	0.124	4.69	z	
	H−4→L+4	29.54				x,y	
	H−4→L+5	14.13				x,y	
**12**	H−8→L	9.18	258.16	0.061	4.80	z	
	H−6→L	38.48				z	

*^a^* Computed at the TDDFT/CAM-B3LYP level of theory using the IEFPCM scheme (solvent = CH_2_Cl_2_) on ground state geometries optimized *in vacuum*; *^b^* Absorption wavelength (*λ* in nm); *^c^* Oscillator strength (*f*_osc_); *^d^* Vertical transition energies (*E* in eV).

**Table 7:** Average polarizability $\bar{\alpha }$ and Δ*α* (in a.u.) of unsubstituted Hückel porphyrinoids computed at different frequencies (*ω* in eV) *^a^*.

System	$\bar{\alpha }$(−*ω*,*ω*)				Δ*α* (−*ω*,*ω*)			
	0	0.413	0.583	0.653	0	0.583	0.653	0.413
**16N** (*C*_2_)	274 (348)	275.5 (325)	277 (327)	277 (353)	237 (329)	239 (301)	242 (304)	243 (339)
**16P** (*S*_4_)	306 (381)	307 (358)	308 (385)	309 (386)	263 (344)	264 (322)	266 (350)	267 (352)
**18P** (*D*_2*h*_)	349 (458)	333 (424)	353 (427)	354 (467)	333 (465)	336 (425)	339 (430)	341 (479)
**18Py** (*C*_2*h*_)	364 (488)	367 (495)	371 (502)	373 (506)	360 (519)	365 (529)	370 (539)	373 (545)
**20P** (*C_i_*)	332 (424)	333 (427)	335 (430)	336 (432)	310 (428)	313 (433)	315 (438)	317 (440)
**20O** (*C*_2*v*_)	368 (472)	370 (476)	373 (480)	374 (483)	354 (497)	358 (553)	361 (504)	363 (508)
**22S** (*C_i_*)	431 (573)	434 (579)	438 (585)	439 (589)	419 (595)	424 (604)	429 (614)	432 (619)
**22I** (*C_s_*)	466 (637)	471 (647)	476 (657)	479 (663)	475 (693)	482 (707)	489 (723)	493 (732)
**22Sp** (*C_s_*)	476 (641)	480 (649)	484 (657)	486 (661)	487 (698)	493 (709)	499 (721)	502 (728)

*^a^*$\bar{\alpha }$(−*ω*,*ω*) and Δ*α* (−*ω*,*ω*) were evaluated at the CAM-B3LYP/6-311+G(d,p) level of theory, both in gas-phase and in solvent (values in parenthesis).

**Table 8:** Hyper-Rayleigh scattering (HRS) first hyperpolarizability characteristics (*β*_HRS_) and depolarization ratio (DR), longitudinal (//) component and second hyperpolarizability (*β*_//_ of unsubstituted Hückel porphyrinoids *^a^*.

System	*β*_HRS_		DR		*β*_//_(−2*ω*,*ω*,*ω*)		*γ*_//_(2*ω*,*ω*,*ω*,0)	
	0	0.653	0	0.653	0	0.653	0	0.653
**16N** (*C*_2_)	115 (211)	119 (226)	1.53 (1.54)	1.15 (1.18)	25 (−53)	−29 (−59)	111 (247)	25 (−1)
**16P** (*S*_4_)	118 (198)	134 (229)	1.50 (1.50)	1.50 (1.50)	0 (0)	0 (0)	162 (355)	204 (461)
**18P** (*D*_2h_)	0 (0)	0 (0)	-*^b^*	-*^b^*	0 (0)	0 (0)	89 (20)	102 (240)
**18Py** (*C_2h_*)	1 (3)	2 (6)	-*^b^*	-*^b^*	2 (4)	2 (8)	−34 (−246)	−94 (−528)
**20P** (*C_i_*)	0 (0)	2 (3)	-*^b^*	-*^b^*	0 (0)	0 (1)	199 (499)	728 (1722)
**20O** (*C*_2v_)	1308 (2982)	23376 *^c^* (86032)	3.67 (3.89)	1.73 *^c^* (1.65)	−1692 (−3956)	−22466 *^c^* (−81580)	254 (649)	5087 *^c^* (37351)
**22S** (*C*_1_)	1281 (3846)	1967 (6307)	6.22 (6.15)	6.18 (6.29)	−1937 (−5762)	−2947 (−9373)	322 (976)	476 (1601)
**22I** (*C*_s_)	1150 (3002)	2255 (6629)	3.41 (2.77)	3.83 (2.90)	−1369 (−3201)	−2783 (−1067)	78 (61)	65 (−122)
**22Sp** (*C_s_*)	516 (1279)	623 (1652)	1.66 (1.78)	1.77 (2.11)	−232 (−686)	310 (−1061)	168 (475)	230 (735)

*^a^β*_HRS_ and *β*_//_(−2*ω*,*ω*,*ω*) (in a.u.) and *γ*_//_(−2*ω*,*ω*,*ω*,0) (in 10^3^ a.u.) were evaluated at the CAM-B3LYP/6-311+G(d,p) level of theory, both in gas-phase and in solvent (values in parenthesis); *^b^* DR is not reported since *β*_HRS_ = 0; *^c^* **20O** is at resonance at 0.653 eV.

**Table 9:** Energetic, structural, magnetic properties of selected *meso*-substituted porphyrinoids *^a^*.

System	π *^b^*	NICS(0)	NICS(1)	NICS_zz_(1)	HOMA	*Π*	Φ_p_	Δ*E*_H-L_
**16N**	16	20.1	16.4	51.9	0.580	0.49	20.4	3.65
**18P**	18	−13.9	−12.8	−35.5	0.860	1.00	1.1	4.59
**20O**	20	17.2	15.1	50.0	0.780	1.00	0.0	3.52
**22S**	22	−9.8	−9.3	−24.7	0.890	0.89	15.0	4.14
**22I**	22	−17.1	−15.0	−40.4	0.888	0.87	12.0	3.79

*^a^* NICS-based indices are in ppm, Φ_p_ in °, and HOMO-LUMO energy difference in eV; *^b^* Number of π-electrons along the classical conjugation pathway.

**Table 10:** *β*_HRS_ and depolarization ratio (DR), longitudinal component of first and second hyperpolarizability (*β*_//_ and *γ*_//_ of *meso*-substituted porphyrinoids in the static and dynamic regime *^a^*.

	*β*_HRS_		DR		*β*_//_(−2*ω*,*ω*,*ω*)		*γ*_//_(−2*ω*,*ω*,*ω*,0)	
*ω*	0	0.653	0	0.653	0	0.653	0	0.653
**16N** (*C*_2_)	3 (6)	6 (12)	-*^b^*	-*^b^*	−1 (−1)	−1 (−1)	375 (749)	229 (376)
**18P** (*D*_2*h*_)	0 (0)	0 (1.0)	-*^b^*	-*^b^*	0 (0)	0 (0)	219 (458)	268 (580)
**20O** (*C*_2*v*_)	1385 (3146)	3373 (6628)	3.64 (3.92)	0.40 (0.27)	1785 (4185)	−1614 (−2007)	413 (964)	−363 (−831)
**22S** (*C*_1_)	2073 (4961)	3448 (8763)	3.50 (3.96)	3.15 (3.69)	−1240 (−5045)	−1810 (−8372)	825 (2078)	1324 (3644)
**22I** (*C_s_*)	2509 (5771)	5191 (13727)	4.14 (3.85)	4.46 (4.07)	3314 (7097)	6725 (16649)	447 (1058)	7325 (1946)

*^a^* All quantities (in a.u. for *β* and 10^3^ a.u. for *γ*) were evaluated at the CAM-B3LYP/6-311+G(d,p) level of theory, both in gas-phase and in solvent (values in parenthesis); *^b^* DR is not reported since *β*_HRS_ = 0.

## Supplementary Materials

Supplementary materials are available online http://www.mdpi.com/1420-3049/23/6/1333/s1.
