# Supplementary material for: Aromaticity as a Guiding Concept for Spectroscopic Features and Nonlinear Optical Properties of Porphyrinoids
Source: Molecules. 2018 Jun 1;23(6):1333. doi: 10.3390/molecules23061333 (PMC6100263; doi:10.3390/molecules23061333)
Supplement: Supplementary file 1 [file molecules-23-01333-s001.pdf]

Supporting information

Aromaticity as a Guiding Concept for Spectroscopic Features and Nonlinear Optical Properties of Porphyrinoids

Tatiana Woller,[a] Paul Geerlings,[a] Frank De Proft,[a] Benoît Champagne[b] and Mercedes Alonso[a*]

[a] *Eenheid Algemene Chemie (ALGC), Vrije Universiteit Brussel (VUB). Pleinlaan 2, 1050 Brussels (Belgium)*

[b] *University of Namur, Laboratoire de Chimie Théorique, Unité de Chimie Physique Théorique et Structurale, rue de Bruxelles 61,B-5000 Namur, Belgium*

E-mail : mercedes.alonso.giner@vub.be

This PDF files includes :

I. Aromaticity descriptors for the Hückel porphyrinoids

II. Geometry and tautomerism of neutral unsubstituted sapphyrin

III. Aromaticity of porphycene

IV. Photophysical properties of unsubstituted porphyrinoids

V. Frequency dispersion of the nonlinear optical properties of unsubstituted porphyrinoids

VI. Cartesian coordinates of M06/6-31G(d,p) optimized geometries

I. Aromaticity descriptors for the Hückel porphyrinoids

Table S1. Energetic, reactivity, magnetic, structural and electronic properties of unsubstituted porphyrinoids.[a]

π e[b]	ISE	ISE_{corr}	$\Delta\eta$	Λ	NICS(0)	NICS(1)	$NICS_{zz}(1)$	HOMA	Π	Φ_p	ΔE_{H-L}[d]	AV1245[e]	AV_{min}[e]
**16N**	20.8	12.4[c]	-3.0	78.8	23.1	18.9	62.3	0.610	0.44	31.7	3.76	1.262	0.136
**16P**	10.4	0.7	-11.9	27.5	9.4	7.6	29.2	0.634	0.74	20.2	4.15	0.732	0.294
**18P**	27.9	11.1	9.2	-175.8	-14.9	-13.7	-38.5	0.880	1.00	0.0	4.75	2.139	1.271
**18Py**	30.9	-0.6[c]	9.8	-147.0	-13.3	-12.5	-13.9	0.878	0.99	0.0	4.07	2.114	1.399
**20P**	12.3	-5.2	-17.8	156.3	18.7	16.7	52.4	0.641	0.93	2.8	3.71	1.226	0.856
**20O**	19.1	17.4[c]	-13.9	128.6	16.3	14.0	44.6	0.780	1.00	0.0	3.27	1.352	0.267
**22S**	40.8	17.2	2.2	-140.6	-9.6	-9.2	-24.6	0.892	0.88	10.3	4.25	2.066	0.794
**22Sp**	25.1	-18.6[c]	13.7	-249.6	-14.3	-13.4	-37.7	0.882	1.00	0.0	4.03	2.124	1.137
**22I**	23.6	6.2	1.9	-249.1	-13.6	-12.8	-35.7	0.901	0.91	8.8	3.85	2.435	0.963

[a] ISE, ISE_{corr} and $\Delta\eta$ are given in kcal mol^{-1}, Λ in ppm cgs and NICS indices in ppm. [b] Number of π-electrons along the classical conjugation pathway. [c] The large flexibility induces large structural changes in the dihydrogen derivative of the methylene adducts during the optimization. [d] HOMO-LUMO energy difference (in eV) evaluated from the CAM-B3LYP single-point calculations. [e] The electronic aromaticity indices were computed along the annulene conjugation pathway.

II. Geometry and tautomerism of neutral unsubstituted sapphyrin

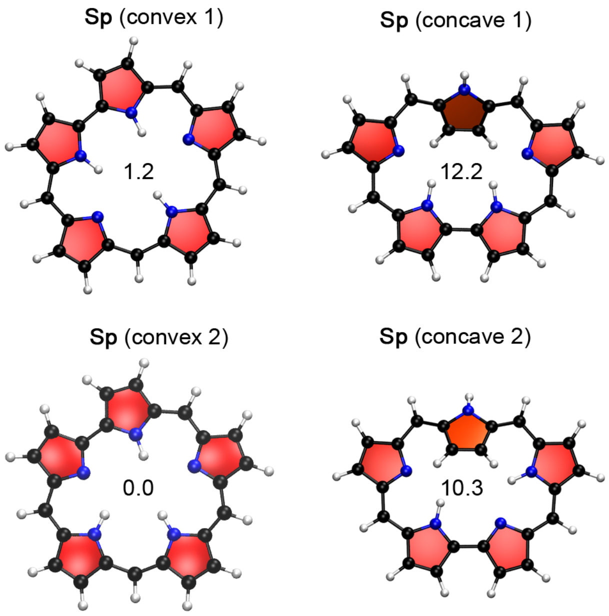

Figure S1. M06/6-311+G(d,p//M06/6-31G(d,p) relative energies for the convex and concave conformations of neutral unsubstituted sapphyrin (**22Sp**). Two different tautomers for each conformation were considered.

III. Aromaticity of porphycene

18Py

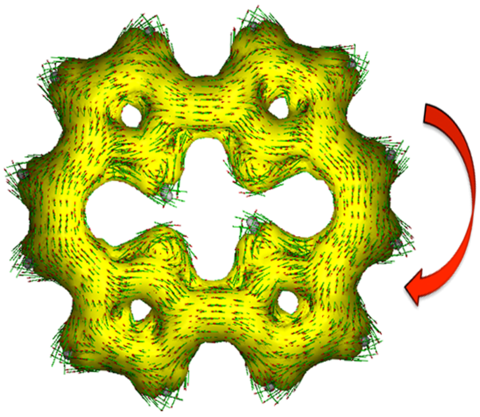

Figure S2. AICD plot of porphycene. The large arrow denotes the direction of the induced ring current: clockwise for diatropic ring currents (isosurface value 0.03 a.u.).

IV. Photophysical properties of unsubstituted porphyrinoids

Table S2. Symmetry of the involved orbitals, associated electronic dipole transition moments of the main electronic transitions of [16]norcorrole (**16N**) calculated at the TDDFT/CAM-B3LYP level of theory using the IEFPCM Scheme (Solvent = CH_2Cl_2) on ground state geometries optimized in vacuum.

excitation	transition	contribution (%)	Irreps initial	Irreps final	μx	μy	μz	polarization	FMO
1	H -> L	98.69	A	A	0.00	0.00	0.01	z	Δk=0
2	H-4 -> L-4	6.96	A	A	0.93	0.47	0.00	z	Δk>1
	H-2 -> L+1	3.33	A	B				x,y	Δk>1
	H-1-> L	84.53	B	A				x,y	Δk=1
3	H-7 -> L	5.70	B	A	0.00	0.00	0.00	x,y	Δk>1
	H-2 -> L	85.09	A	A				z	Δk>1
	H-1 -> L+1	4.69	B	B				z	Δk>1
4	H-4 ->L	2.11	A	A	2.26	-0.32	0.00	z	Δk>1
	H- > L+2	94.22	A	A				z	Δk>1
5	H-5 -> L+2	2.51	B	A	-0.04	-0.37	0.00	x,y	Δk>1
	H-4-> L	2.36	A	A				z	Δk>1
	H-3 -> L	90.21	B	A				x,y	Δk>1
7	H -> L+4	41.38	A	A	-1.80	-0.37	-0.00	z	Δk>1
10	H-4 -> L	81.08	A	A	1.11	-1.60	0.00	z	Δk>1
13	H -> L+4	81.19	A	A	-0.03	2.00	0.00	z	Δk>1

Table S3. Symmetry of the involved orbitals, associated electronic dipole transition moments of the main electronic transitions of [18]porphyrin (**18P**) calculated at the TDDFT/CAM-B3LYP level of theory using the IEFPCM Scheme (Solvent = CH_2Cl_2) on ground state geometries optimized in vacuum.

Excitation	transition	contribution (%)	Irreps initial	Irreps final	μx	μy	μz	polarization	FMO
1	H-1 -> L	47.52	Au	B3g	0.12	0	0	x	Δk=1
	H -> L+1	52.62	B1u	B2g				x	Δk =1
2	H-1 -> L+1	51.15	Au	B2g	0	-0.18	0	y	Δk =1

	H -> L	48.60	B1u	B3g				y	Δk =1
3	H-4 -> L+1	12.58	B1u	B2g	3.27	0	0	x	Δk >1
	H-1 -> L	48.22	Au	B3g				x	Δk =1
	H -> L+1	39.86	B1u	B2g				x	Δk =1
	L+1 -> H	2.46	B2g	B1u				x	Δk =1
4	H-1 -> L+1	49.12	Au	B2g	0	-3.65	0	y	Δk =1
	H -> L	52.30	B1u	B3g				y	Δk =1
5	H-3 -> L+2	4.98	B2g	Au				/	Δk =1
	H-2 -> L+1	91.50	B3g	B2g				/	Δk =1
	H -> L+2	2.08	Au	A1u	-0.01	0.00	0.00	z	Δk >1
6	H-5 -> L	80.03	B3g	B2g				y	Δk >1
16	H-5 -> L	85.33	B1u	Au				y	Δk >1
18	H-4 -> L	96.34	B1u	B3g	0.46	2.10	0	x	Δk >1

Table S4. Symmetry of the involved orbitals, associated electronic dipole transition moments of the main electronic transitions of [20]orangarin (**20O**) calculated at the TDDFT/CAM-B3LYP level of theory using the IEFPCM Scheme (Solvent = CH_2Cl_2) on ground state geometries optimized in vacuum.

xcitation	transition	contribution (%)	Irreps initial	Irreps final	μx	μy	μz	polarization	FMO
1	H -> L	97.55	A2	B1	0.00	0.47	0.00	y	Δk=0
2	H-1 -> L+1	98.45	A2	A2	0.00	0.00	-2.89	z	Δk=1
3	H-1 -> L	6.86	A2	B1	0.00	1.54	0.00	y	Δk=1
	H-4 -> L	83.88	A2	B1				y	Δk=1
4	H-3 -> L	-42.60	B1	B1	0.00	0.00	1.32	z	Δk>1
	H-2 -> L	28.92	B1	B1				z	Δk=1
5	H-3 -> L	27.26	B1	B1	0.00	0.00	2.00	z	Δk>1
	H-2 -> L	66.51	B1	B1				z	Δk=1
6	H-4 -> L	67.52	A2	B1	0.00	-1.81	0.00	y	Δk>1
12	H -> L+2	93.84	A2	B1	0.00	-2.40	0.00	y	Δk>1

Table S5. Associated electronic dipole transition moments of the main electronic transitions of [22]smaragdryin (**22S**) calculated at the TDDFT/CAM-B3LYP level of theory using the IEFPCM Scheme (Solvent = CH_2Cl_2) on ground state geometries optimized in vacuum.

excitation	transition	contribution (%)	μx	μy	μz	polarization	FMO
1	H-1 -> L	52.81	0.16	-0.35	-0.00	y	Δk=1
	H -> L+1	42.44					Δk=1
2	H-1 -> L+1	27.12	0.97	-0.18	0.10	x	Δk=1
	H -> L	68.23					Δk=1
3	H-1 -> L	44.57	0.25	4.18	0.01	y	Δk=1
	H -> L+1	55.86					Δk=1
4	H-1 -> L+1	69.40	3.73	-0.23	-0.02	x	Δk=1
	H -> L	28.69					Δk=1
5	H-3 -> L	9.47	-0.90	-0.09	-0.00	x	Δk>1
	H-2 -> L	77.50					Δk>1
8	H -> L+3	61.17	-0.91	-0.04	-0.01	x	Δk>1
	H-1 -> L+6	9.28					Δk>1
11	H-5 -> L	24.76	0.51	0.37	-0.07	x	Δk>1
	H-2 -> L+1	11.93					Δk>1
	H-9 -> L	9.68					Δk>1
	H-1 -> L+4	15.21					Δk>1

Table S6. Symmetry of the involved orbitals, associated electronic dipole transition moments of the main electronic transitions of [22]isosmaragdyrin (**22I**) calculated at the TDDFT/CAM-B3LYP level of theory using the IEFPCM Scheme (Solvent = CH_2Cl_2) on ground state geometries optimized in vacuum.

excitation	transition	contribution (%)	Irreps initial	Irreps final	μx	μy	μz	polarization	FMO
1	H-1 -> L+1	28.61	A''	A'	0.00	0.00	-1.50	z	Δk=1
	H -> L	70.93	A'	A''				z	Δk=1
2	H-1 -> L	72.54	A''	A''	1.42	0.01	0.00	x,y	Δk=1
	H -> L+1	26.94	A'	A'				x,y	Δk=1
3	H-1 -> L	28.57	A''	A''	-4.04	-0.04	0.00	x,y	Δk=1
	H -> L+1	73.62	A'	A'				x,y	Δk=1
4	H-1 -> L+1	69.35	A''	A'	0.00	0.00	-3.81	z	Δk=1
	H -> L	28.97	A'	A''				z	Δk=1
5	H-2 -> L	93.02	A'	A''	0.00	0.00	-1.38	z	Δk>1
	H-1 -> L+1	2.46	A''	A'				z	Δk=1
9	H-4 -> L	29.11	A'	A''	0.00	0.00	1.04	z	Δk>1
	H-4 -> L+4	29.54	A'	A'				x,y	Δk>1
	H-4 -> L+5	14.13	A'	A'				x,y	Δk>1
12	H-8 -> L	9.18	A'	A''	0.00	0.00	0.72	z	Δk>1
	H-6 -> L	38.48	A'	A''				z	Δk>1

Table S7. Properties of the main electronic transitions of neutral unsubstituted [22]sapphyrin (**22Sp**) calculated at the TDDFT/CAM-B3LYP level of theory using the IEFPCM Scheme (Solvent = CH_2Cl_2) on ground state geometries optimized in vacuum.

excitation	transition	contribution(%)	λ^{a}	f_{osc}^{b}	E^{c}	polarization	assignement
**1**	**H → L**	31.19	648.88	0.008	1.91	y	Q
	H → L+1	24.82					
	H-1 → L	23.46					
	H-1 → L+1	20.93					
**2**	**H → L**	29.26	609.01	0.013	2.04	x	Q
	H-1 → L	26.15					
	H → L+1	25.86					
	H-1 → L+1	18.74					
**3**	**H-1 → L**	51.41	379.57	1.468	3.27	y	B
	H → L+1	49.66					
**4**	**H-2 → L**	59.72	369.48	1.625	3.36	x	B
	H → L	39.73					
**5**	**H-3 → L**	37.76	311.63	0.160	3.98	x	
	H-2→ L	31.88					
	H-3 → L+1	8.98					
**6**	**H-2 → L+1**	51.32	306.47	0.070	4.05	y	
	H-2 → L	15.45					
	H-3 → L+1	13.04					
**8**	**H-2 → L**	29.13	279.85	0.067	4.43	x	
	H → L+2	19.33					
	H-3 → L	15.57					
	H-2 → L+1	13.21					

[a] Absorption wavelength (λ in nm). [b] Oscillator strength (f_{osc}). [c] Vertical transition energies (E in eV).

Table S8. Properties of the main electronic transitions of neutral unsubstituted [18]porphycene (**18Py**) calculated at the TDDFT/CAM-B3LYP level of theory using the IEFPCM Scheme (Solvent = CH_2Cl_2) on ground state geometries optimized in vacuum.

excitation	transition	contribution(%)	λ^a	$f_{osc}{}^b$	E^c	polarization	assignement	FMO
**1**	**H → L**	79.76	570.98	0.161	2.17	x	Q	Δk=1
	H → L+1	17.70						Δk=1
**2**	**H-1 → L+1**	83.93	542.80	0.237	2.28	y	Q	Δk=1
	H → L	14.16						Δk=1
**3**	**H-2 → L**	96.45	351.00	0.000	3.53	x		Δk>1
**4**	**H-2 → L**	80.48	350.13	0.027	3.54	x		Δk>1
	H → L+1	15.71						Δk=1
**5**	**H-1 → L+1**	64.68	315.98	0.785	3.92	y	B	Δk=1
	H → L	15.46						Δk=1
	H → L+1	10.96						Δk=1
**6**	**H → L+1**	44.79	309.66	0.877	4.00	x	B	Δk=1
	H-1 → L+1	15.45						Δk=1
	H-4 → L+1	14.89						Δk>1
**9**	**H-4 → L**	80.87	286.46	0.420	4.33	x		Δk>1
	H-1 → L+1	6.08						Δk=1

[a] Absorption wavelength (λ in nm). [b] Oscillator strength (f_{osc}).[c] Vertical transition energies (E in eV).

Table S9. Properties of the main electronic transitions of neutral unsubstituted [16]porphyrin (**16P**) calculated at the TDDFT/CAM-B3LYP level of theory using the IEFPCM Scheme (Solvent = CH_2Cl_2) on ground state geometries optimized in vacuum.

excitation	transition	contribution(%)	λ^a	$f_{osc}{}^b$	E^c	polarization	assignement	FMO
**1**	**H → L**	97.77	761.93	0.000	1.63	/	Q	Δk=0
**2**	**H-1→ L**	84.33	527.32	0.000	2.35	/		Δk=1
	H-5 → L	7.40						Δk>1
**3**	**H-7 → L**	43.29	418.24	0.008	2.96	x		Δk>1
	H-2 → L	21.64						Δk=1
	H-3 → L	11.77						Δk=1
**4**	**H-6→ L**	43.32	418.24	0.008	2.96	y		Δk>1
	H-3 → L	21.61						Δk=1
	H-2→ L	11.77						Δk=1
**5**	**H-4→ L**	89.62	415.69	0.000	2.98	z		Δk>1
	H-3→ L+2	3.12						Δk>1
	H-2 → L+1	3.12						Δk>1
**10/11**[d]	**H → L+1**	75.85	316.08	0.166	3.92	y/x		Δk=1
	H -8→ L	16.54						Δk>1
**12/13**[d]	**H-8 → L**	58.04	293.76	0.677	4.22	y/x	B	Δk>1
	H → L+1	9.85						Δk=1
	H-2 → L	6.95						Δk=1
**18/19**[d]	**H-4→ L+1**	62.97	248.08	0.176	4.99	x/y		Δk>1
	H-4→ L+2	7.67						Δk>1
	H-2→ L+3	6.48						Δk>1

[a] Absorption wavelength (λ in nm). [b] Oscillator strength (f_{osc}).[c] Vertical transition energies (E in eV). [d] Due to the S4 symmetry, there are degenerated orbitals and thus some electronic transition with the same excitation energy, wavelength and oscillator strength.

Table S10. Properties of the main electronic transitions of neutral unsubstituted [20]porphyrin (**20P**) calculated at the TDDFT/CAM-B3LYP level of theory using the IEFPCM Scheme (Solvent = CH_2Cl_2) on ground state geometries optimized in vacuum.

excitation	transition	contribution(%)	λ^a	f_{osc}^b	E^c	polarization	assignement	FMO
**1**	**H → L**	98.22	928.99	0.000	1.33	/	Q	Δk=0
**2**	**H → L+1**	94.59	361.36	0.466	3.43	x		Δk=1
	H-2 → L	3.74						Δk=1
**3**	**H → L+2**	88.83	340.02	0.000	3.66	/		Δk>1
	H → L+8	3.81						Δk>1
**4**	**H-1 → L**	81.74	326.41	0.231	3.80	y		Δk=1
	H → L+3	9.92						Δk>1
**5**	**H-2 → L**	93.97	306.21	0.964	4.05	x	B	Δk=1
	H → L+1	3.98						Δk=1
**6**	**H → L+3**	71.42	302.43	0.518	4.10	y		Δk>1
	H-1 → L	13.52						Δk=1
	H → L+4	5.45						Δk>1
**7**	**H-4 → L**	79.78	285.29	0.264	4.35	y		Δk=1
	H → L+3	5.37						Δk>1
	H-2 → L+2	3.52						Δk>1

[a] Absorption wavelength (λ in nm). [b] Oscillator strength (f_{osc}). [c] Vertical transition energies (E in eV).

V. Frequency dispersion of the nonlinear optical properties of unsubstituted porphyrinoids

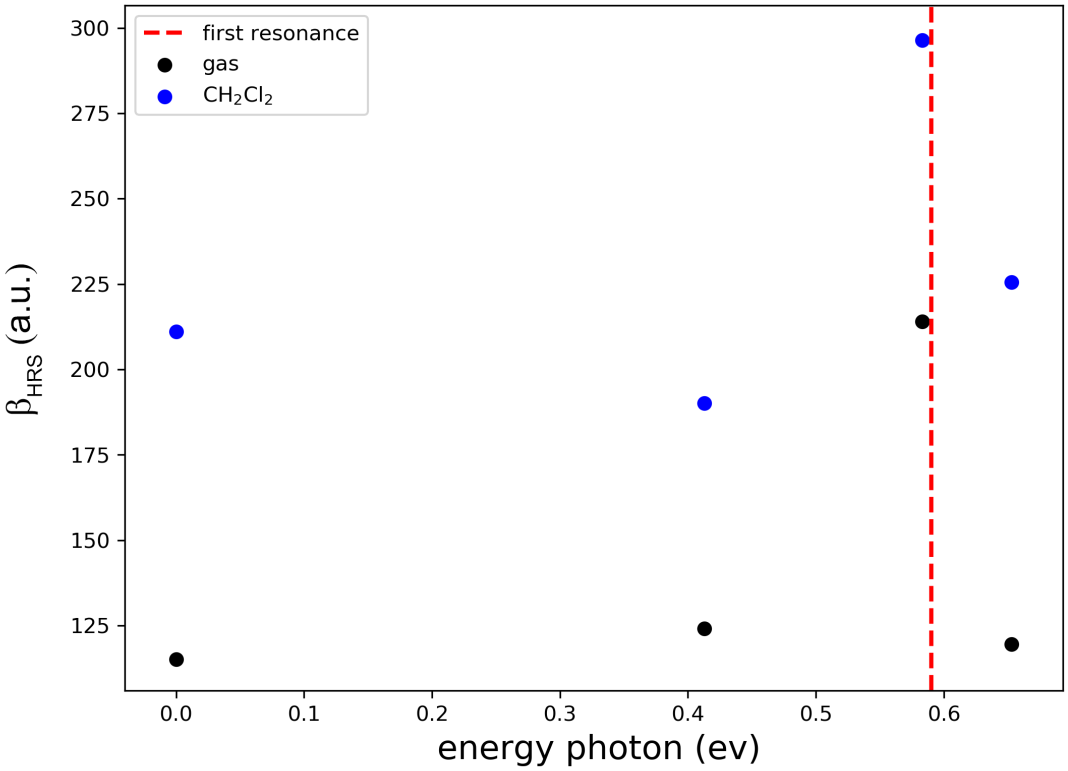

Figure S3. Frequency dispersion of β_{HRS} in **16N**.

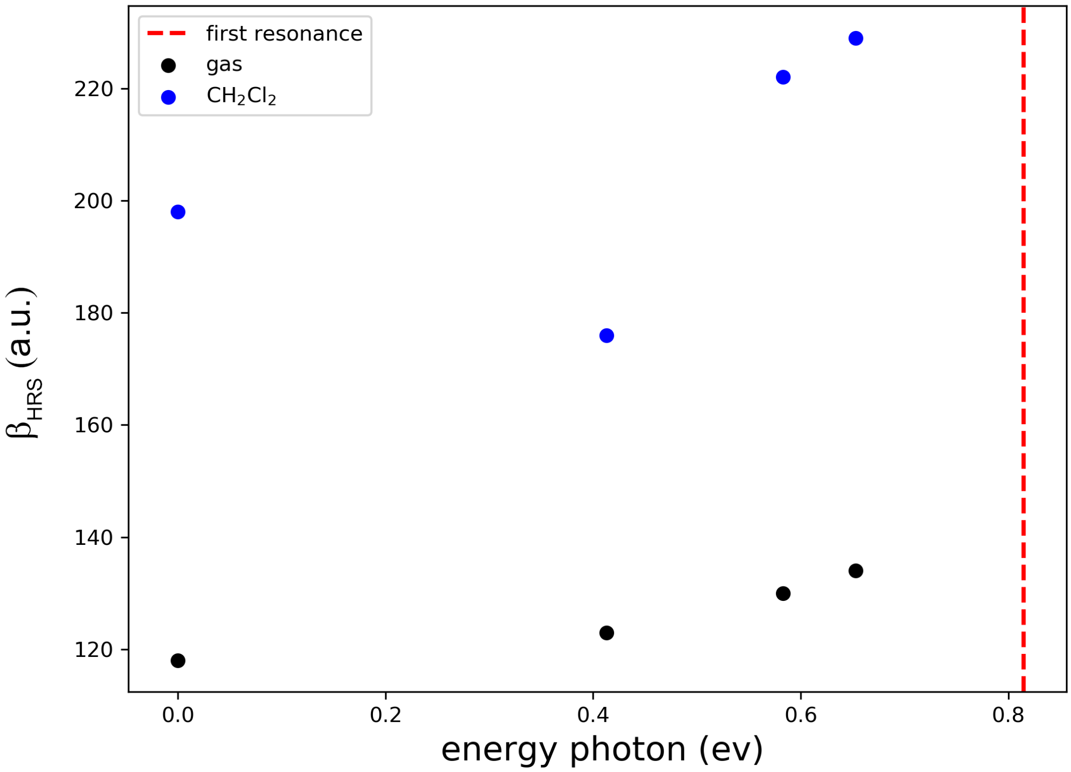

Figure S4. Frequency dispersion of β_{HRS} in **16P**.

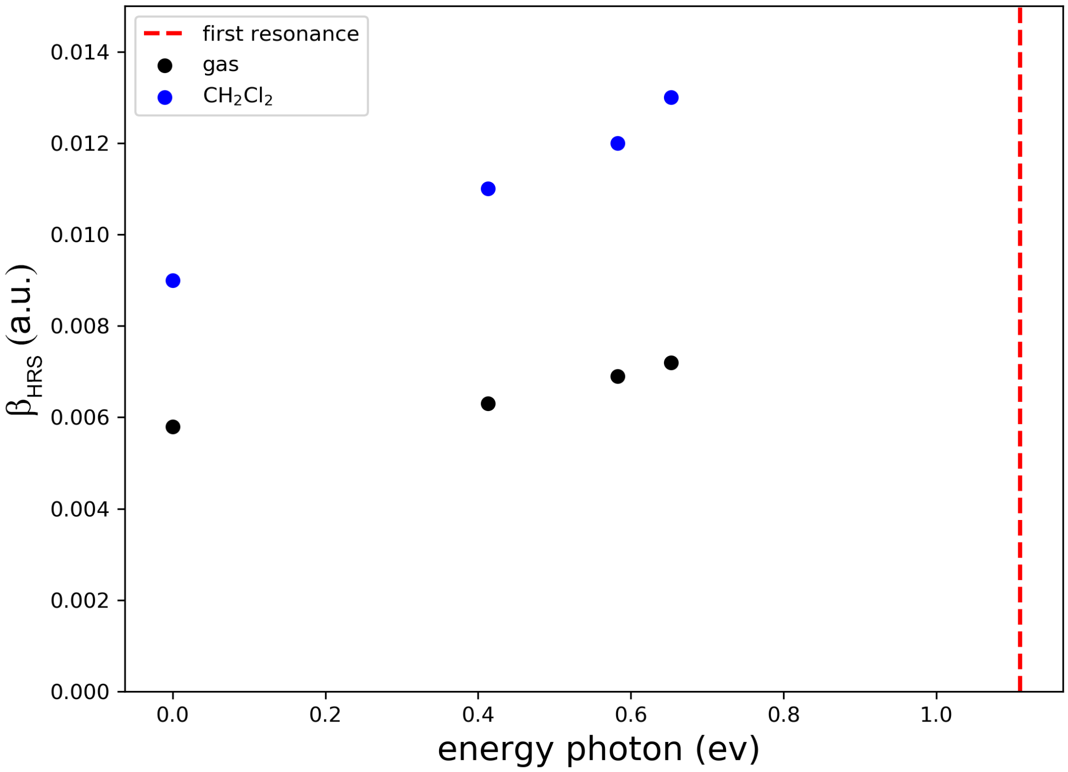

Figure S5. Frequency dispersion of β_{HRS} in **18P**.

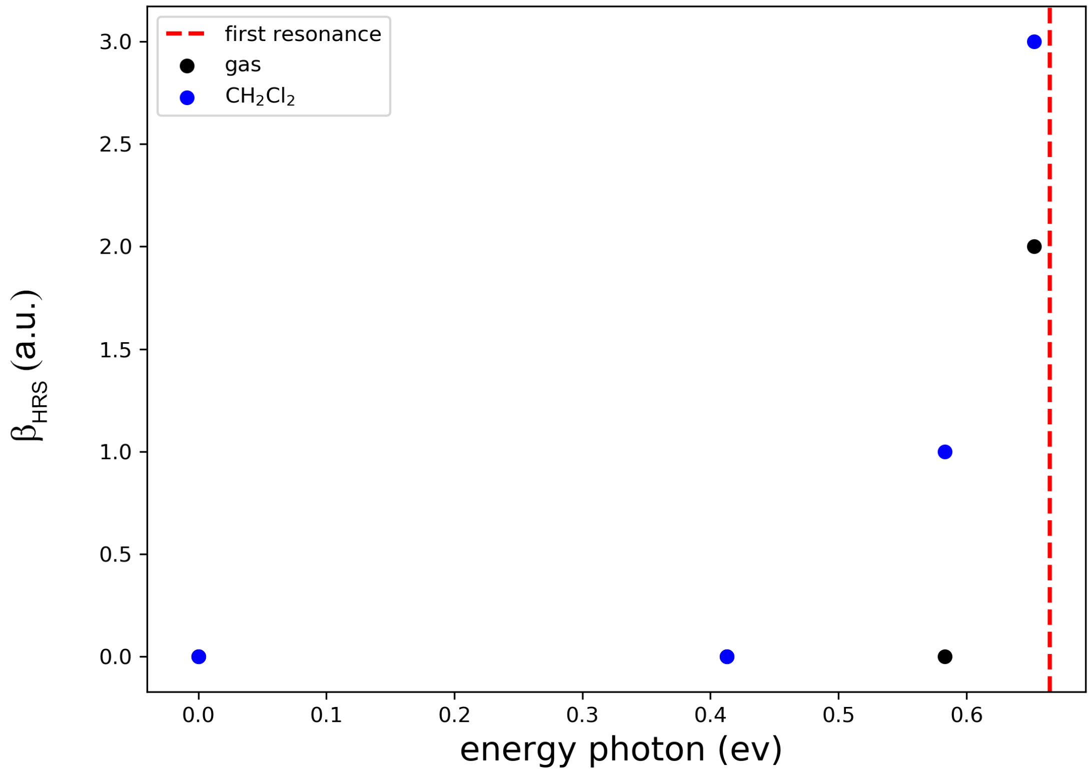

Figure S6. Frequency dispersion of β_{HRS} in **20P**.

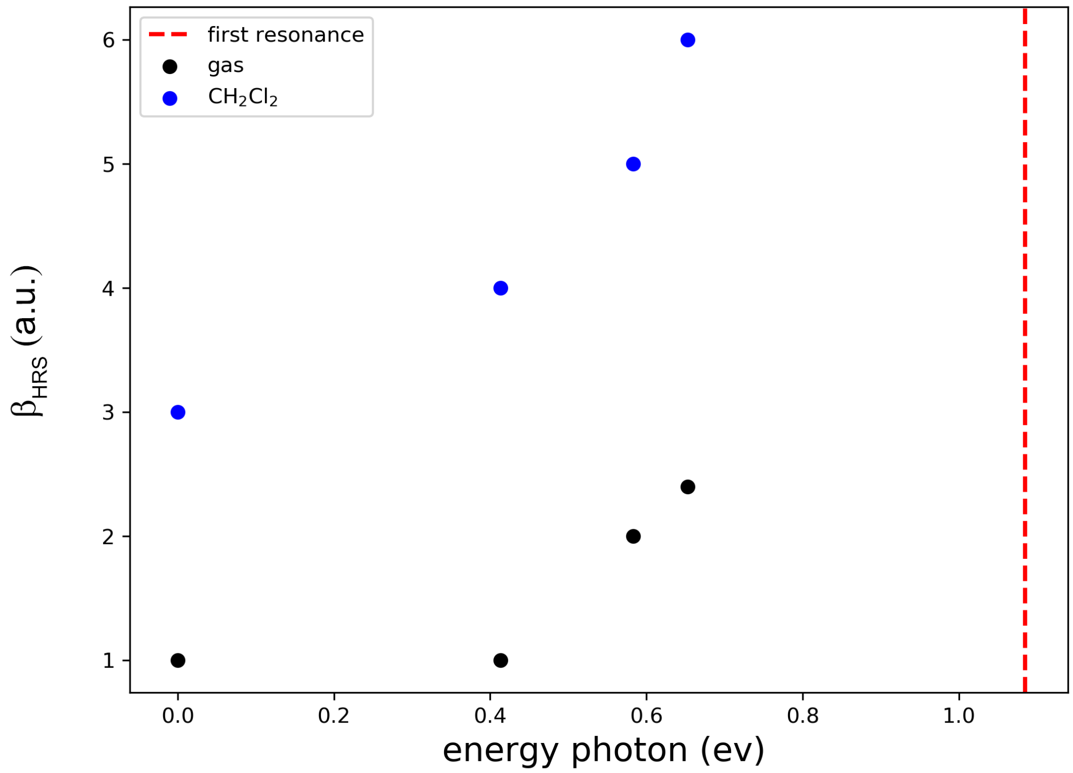

Figure S7. Frequency dispersion of β_{HRS} in **18Py**.

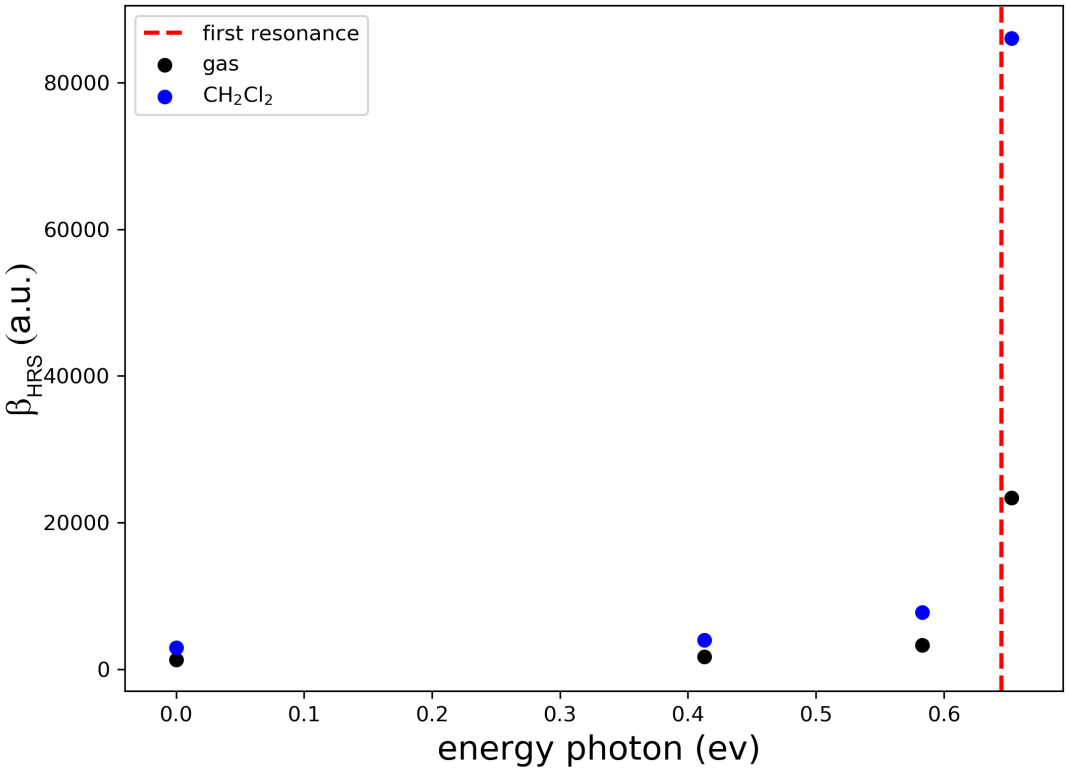

Figure S5. Frequency dispersion of β_{HRS} in **20O**.

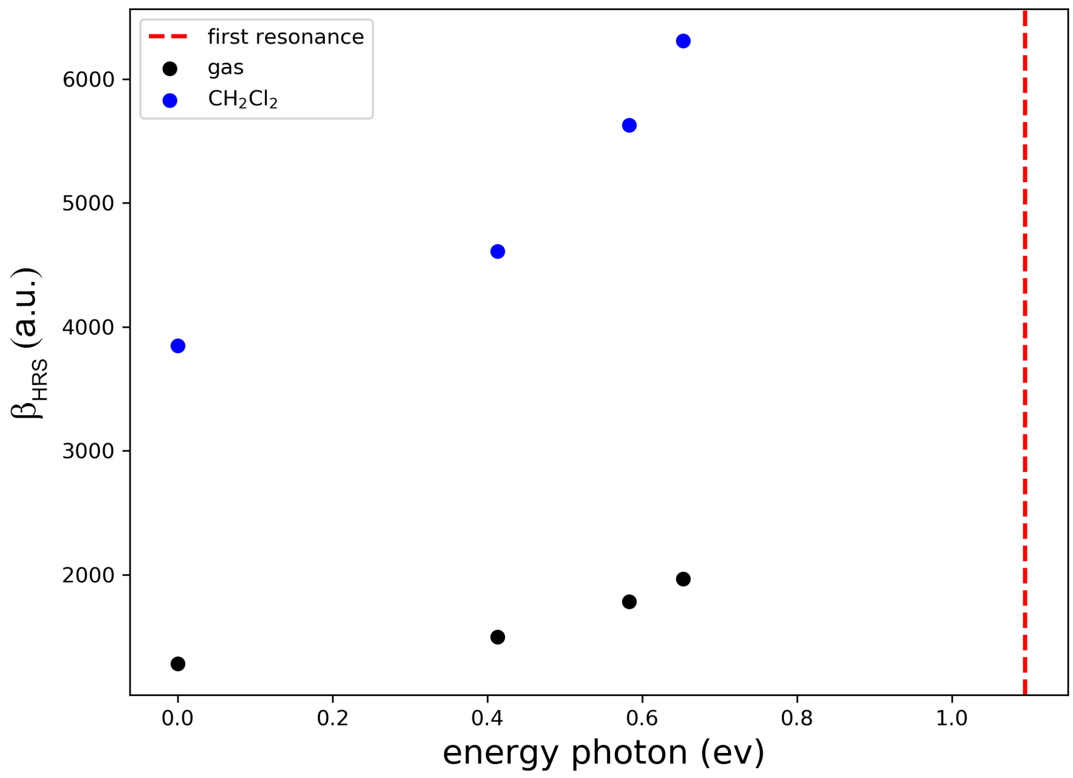

Figure S6. Frequency dispersion of β_{HRS} in **22S**.

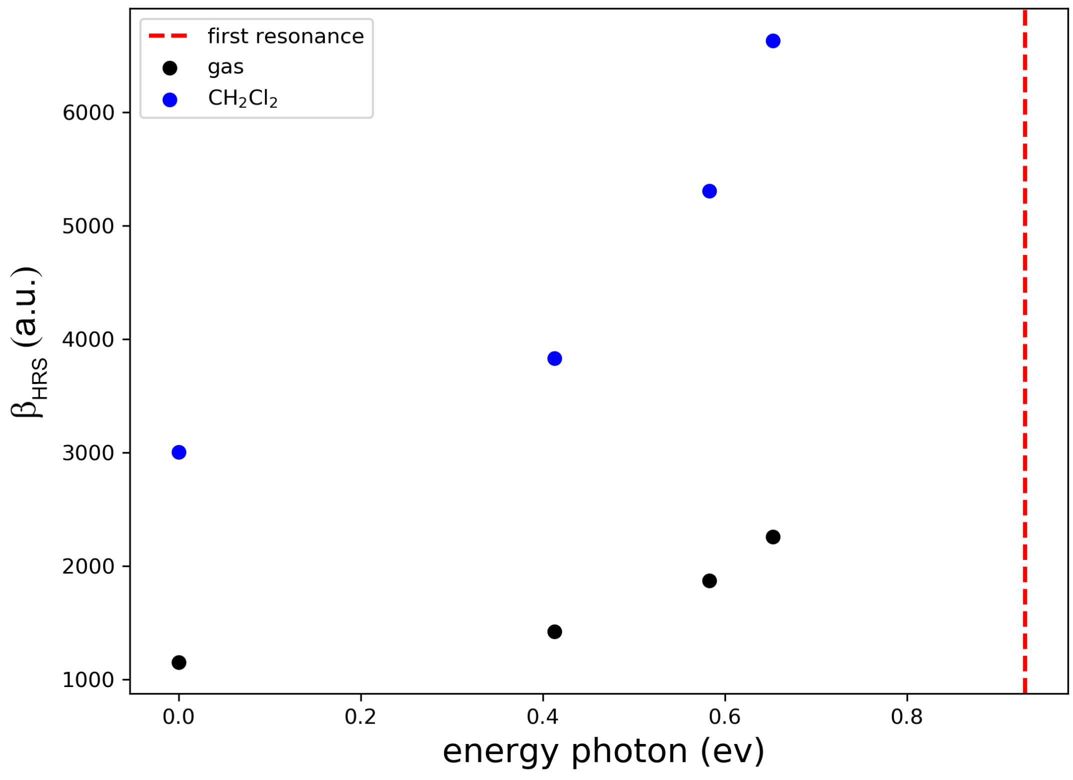

Figure S7. Frequency dispersion of β_{HRS} in **22I**.

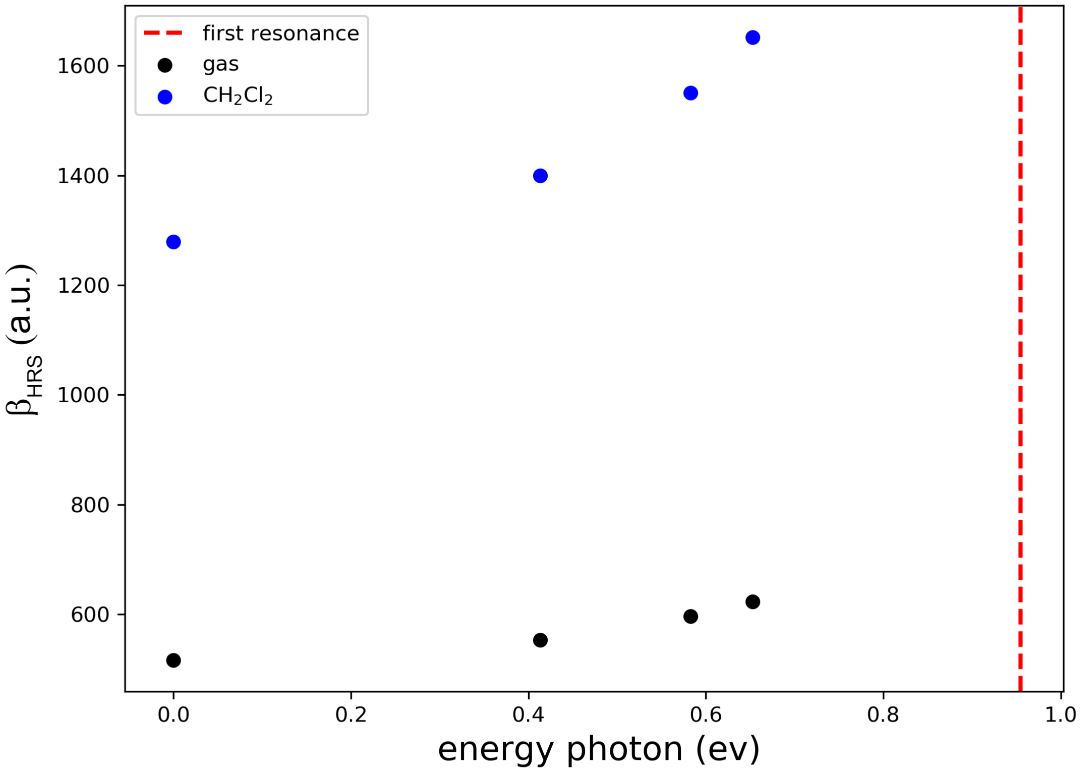

Figure S8. Frequency dispersion of β_{HRS} in **22Sp**.

Table S11. β_{HRS} and depolarization ratio (DR) of unsubstituted Hückel porphyrinoids computed at different wavelength using CAM-B3LYP/6-311+g(d,p) in gas-phase and in (solvent).

	β_{HRS}(-2ω,ω,ω)				DR			
**ω**	**0**	**0.413**	**0.583**	**0.653**	**0**	**0.413**	**0.583**	**0.653**
**16N** (*C*2)	115.1 (211.1)	124.1 (190.2)	214.0 (296.5)	119.5 (225.6)	1.53 (1.54)	1.52 (1.59)	0.96 (1.07)	1.15 (1.18)
**16P** (*S*4)	118 (198)	123 (176)	130 (222)	134 (229)	1.50 (1.50)	1.50 (1.50)	1.50 (1.50)	1.50 (1.50)
**18P** (*D*2h)	0.0 (0.0)	0.0 (0.0)	0.0 (0.0)	0.0 (0.0)	-[a]	-[a]	-[a]	-[a]
**18Py** (*C*2*h*)	1 (3)	1 (4)	2 (5)	2 (6)	-[a]	-[a]	-[a]	-[a]
**20P** (*C**i*)	0 (0)	0 (0)	0 (1)	2 (3)	-[a]	-[a]	-[a]	-[a]
**20O** (*C*2v)	1307.9 (2981.7)	1744.7 (4021.7)	3309.4 (7804.9)	23375.9 (86032.0)	3.67 (3.89)	3.70 (3.93)	3.22 (3.34)	1.73 (1.65)
**22S** (*C*1)	1280.5 (3846.3)	1498.4 (4607.9)	1782.5 (5628.5)	1967.1 (6306.6)	6.22 (6.15)	6.20 (6.19)	6.19 (6.25)	6.18 (6.29)
**22I** (*C*s)	1149.9 (3002.2)	1419.8 (3830.5)	1871.9 (5304.1)	2255.3 (6628.6)	3.41 (2.77)	4.12 (3.26)	4.22 (3.23)	3.83 (2.90)
**22Sp** (*C**s*)	516 (1279)	553 (1400)	596 (1551)	623 (1652)	1.66 (1.78)	1.71 (1.89)	1.76 (2.03)	1.77 (2.11)

[a] DR is not reported since β_{HRS} equals 0.

Table S12. Longitudinal component of first and second hyperpolarizability of unsubstituted Hückel porphyrinoids computed at different wavelength using CAM-B3LYP/6-311+g(d,p) in gas-phase and in (solvent).

	β//(-2ω,ω,ω)				γ//(-2ω,ω,ω,0)			
System	**0**	**0.413**	**0.583**	**0.653**	**0**	**0.413**	**0.583**	**0.653**
**16N** (C_2)	24.8 (-52.8)	24.6 (-68.9)	9.96 (-42.9)	-28.9 (-59.28)	111 (247)	136 (287)	447 (1021)	25 (-1)
**16P** (S_4)	0 (0)	0 (0)	0 (0)	0 (0)	162 (355)	175 (321)	192 (430)	204 (461)
**18P** (D_{2h})	0.0 (0.0)	0.0 (0.0)	0.0 (0.0)	0.0 (0.0)	89 (20)	95 (195)	99 (207)	102 (240)
**18Py** (C_{2h})	0 (4)	2 (5)	2 (7)	1 (8)	-34 (-246)	-51 (-326)	-76 (-444)	-94 (-528)
**20P** (C_i)	0 (0)	0 (0)	0 (0)	0 (1)	199 (499)	230 (586)	306 (798)	728 (1722)
**20O** (C_{2v})	-1691.9 (-3956.3)	-2188.9 (-5181.1)	-3828.1 (-9208.8)	-22466.4 (-81579.9)	254 (649)	330 (883)	608 (1796)	5087[c] (37351)
**22S** (C_1)	-1937.5 (-5762.1)	-2258.7 (-6880.7)	-2676.3 (-8379.1)	-2946.8 (-9372.9)	322 (976)	372 (1168)	435 (1426)	476 (1601)
**22I** (C_s)	-1368.9 (-3200.9)	-1747.8 (-4283.6)	-2329.2 (-6047.1)	-2783.1 (-1067.0)	78 (61)	79 (29)	73 (-48)	65 (-122)
**22Sp** (C_s)	-231 (-686)	-260 (-806)	-293 (-961)	230 (-1061)	168 (475)	188 (554)	213 (660)	230 (735)

III. Cartesian coordinates of optimized structures at M06/6-31G(d,p) level of theory

1) Unsubstituted [16]norcorrole

```
C                    -2.65066  -2.81561  -0.42107
 H                    -2.99782  -3.75725  -0.82865
 C                     2.43604  -3.03841  -0.2866
 H                     2.71145  -4.03831  -0.60296
 C                     3.23234  -1.92455  -0.28021
 H                     4.26849  -1.85386  -0.58918
 C                    -2.43587   3.03801  -0.28741
 H                    -2.71087   4.0377   -0.60478
 C                    -2.52428  -0.63211   0.16723
 C                    -3.36779  -1.60281  -0.39186
 H                    -4.36835  -1.43795  -0.77085
 C                     0.10671   3.23914   0.0052
 C                     2.65081   2.81597  -0.42063
 H                     2.99803   3.7578   -0.82772
 C                     3.36799   1.60325  -0.39169
 H                     4.36859   1.43863  -0.77065
 C                    -1.12079   2.61506   0.14864
 C                    -1.37189  -2.56838   0.11372
 C                    -0.10693  -3.23919   0.00492
 C                    -2.39573   0.8368    0.18181
 C                    -3.232     1.92405  -0.28125
 H                    -4.26777   1.85287  -0.59137
```

C	1.1206	-2.61503	0.14772
C	2.5244	0.63214	0.1666
C	2.39573	-0.83683	0.18113
C	1.37181	2.56844	0.11352
N	1.19008	-1.27063	0.47547
N	1.38982	1.26843	0.51928
N	-1.38997	-1.26863	0.52038
N	-1.19055	1.27096	0.47758
H	0.51443	0.80759	0.76436
H	-0.51429	-0.80761	0.76423
H	-0.11983	-4.26729	-0.35485
H	0.11939	4.26701	-0.35523

2) Unsubstituted [18]porphyrin

H	-0.00001900	0.00004000	1.10274000
H	-0.00001900	0.00004000	-1.10274000
C	-0.00004900	-4.25641000	0.67807000
C	-0.00004900	-4.25641000	-0.67807000
N	0.00000000	0.00001000	-2.11720000
N	0.00000000	0.00001000	2.11720000
N	0.00000000	2.02784000	0.00000000
C	-0.00005900	-2.85394000	1.08463000
C	-0.00005900	-2.85394000	-1.08463000
N	0.00002100	-2.02784000	0.00000000
C	-0.00003900	-2.44007000	-2.42189000
C	-0.00003900	-2.44007000	2.42189000
C	0.00000000	-1.12951000	-2.89620000
C	0.00000000	-1.12951000	2.89620000
C	0.00004100	-0.68602000	-4.26071000
C	0.00004100	-0.68602000	4.26071000
C	0.00005100	0.68601000	-4.26072000
C	0.00005100	0.68601000	4.26072000
C	0.00002100	1.12952000	-2.89623000
C	0.00002100	1.12952000	2.89623000
C	0.00001100	2.44007000	-2.42189000
C	0.00001100	2.44007000	2.42189000
C	0.00000000	2.85393000	-1.08463000
C	0.00000000	2.85393000	1.08463000
C	0.00000000	4.25640000	-0.67807000
C	0.00000000	4.25640000	0.67807000
H	0.00001100	-3.21846000	-3.17876000
H	0.00001100	-3.21846000	3.17876000
H	0.00006100	-1.34686000	-5.11658000
H	0.00006100	-1.34686000	5.11658000
H	-0.00006900	-5.10404000	1.35146000
H	-0.00006900	-5.10404000	-1.35146000
H	0.00007100	1.34684000	-5.11661000
H	0.00007100	1.34684000	5.11661000
H	0.00002100	3.21847000	-3.17875000
H	0.00002100	3.21847000	3.17875000
H	0.00000000	5.10404000	-1.35145000
H	0.00000000	5.10404000	1.35145000

3) Unsubstituted [20]orangarin

C	0.	0.72133	-2.92667
C	0.	-0.72133	-2.92667
C	0.	-1.62376	-3.99572
C	0.	-2.91491	-3.45848
C	0.	-2.79875	-2.0637
C	0.	-3.79086	-1.04497
C	0.	-3.54236	0.30576
N	0.	-2.25051	0.80966
C	0.	-2.35989	2.1276
C	0.	-1.12612	2.88854
C	0.	-0.70778	4.21781
C	0.	0.70778	4.21781
C	0.	1.12612	2.88854
C	0.	2.35989	2.1276
N	0.	2.25051	0.80966
C	0.	3.54236	0.30576
C	0.	3.79086	-1.04497
C	0.	2.79875	-2.0637
C	0.	2.91491	-3.45848
C	0.	1.62376	-3.99572
N	0.	1.45121	-1.77996
C	0.	-4.48258	1.40373
C	0.	-3.74285	2.54939
N	0.	-1.45121	-1.77996
N	0.	0.	2.13314
C	0.	3.74285	2.54939
C	0.	4.48258	1.40373
H	0.	4.82683	-1.38088
H	0.	-4.82683	-1.38088
H	0.	-1.16706	-0.79903
H	0.	-5.56276	1.31052
H	0.	-4.09708	3.5734
H	0.	-1.35257	5.08766
H	0.	1.35257	5.08766
H	0.	4.09708	3.5734
H	0.	5.56276	1.31052
H	0.	3.84948	-4.00554
H	0.	1.35068	-5.04308
H	0.	-1.35068	-5.04308
H	0.	-3.84948	-4.00554
H	0.	1.16706	-0.79903
H	0.	0.	1.1265

4) Unsubstituted [22]smaragdyrin

N	-2.05256	1.32949	-0.0623
C	-1.79564	2.63669	0.05432
C	-0.46702	3.1241	0.14911
C	0.03854	4.41248	0.35364
C	1.42634	4.33467	0.32444
C	1.78975	2.99542	0.10134
C	3.08595	2.46357	-0.04455

C	3.48494	1.15407	-0.14659
C	4.80997	0.69582	-0.41098
C	4.8202	-0.66667	-0.39237
C	3.50113	-1.1355	-0.11661
C	3.11544	-2.44635	0.01746
C	1.81998	-2.98241	0.15765
C	1.45369	-4.30757	0.42819
C	0.0646	-4.39245	0.40875
C	-0.44123	-3.12282	0.12523
C	-1.78068	-2.68407	0.00007
C	-2.97276	-3.43301	-0.08142
C	-4.02418	-2.53854	-0.20331
C	-3.48856	-1.2343	-0.19348
C	-4.10399	0.02398	-0.22479
C	-3.42539	1.23008	-0.14522
C	-4.02395	2.5399	-0.08702
H	-5.08746	2.7466	-0.13384
C	-2.99987	3.4282	0.04672
H	-3.05508	4.50803	0.12165
H	-0.56402	5.29635	0.52072
H	2.13367	5.14335	0.46199
H	-0.5409	-5.26688	0.61174
H	5.64203	1.35944	-0.61464
H	5.66165	-1.32354	-0.5782
H	2.15616	-5.10511	0.63615
H	-3.02736	-4.51426	-0.06958
H	-5.07748	-2.77424	-0.29228
N	0.61345	2.28063	0.02886
H	0.51518	1.34556	-0.33953
N	0.64187	-2.26661	0.01379
H	0.6095	-1.39595	-0.50096
N	-2.12264	-1.36929	-0.08249
H	-1.56261	-0.51827	0.02549
N	2.67781	0.00461	-0.02385
H	1.95101	0.00594	0.68502
H	3.88572	3.20004	-0.09113
H	3.92129	-3.1772	0.00139
H	-5.19031	0.04669	-0.28553

5) Unsubstituted [22]isosmaragdyrin

C	0.00337	-0.57312	3.65982
C	-0.16382	-1.71804	4.47775
C	-0.13457	-2.84381	3.67152
C	0.05893	-2.41423	2.34298
C	0.11739	-3.03308	1.07535
N	0.19487	-2.21822	0.
C	0.11739	-3.03308	-1.07535
C	0.05893	-2.41423	-2.34298
C	-0.13457	-2.84381	-3.67152
C	-0.16382	-1.71804	-4.47775
C	0.00337	-0.57312	-3.65982
C	-0.01484	0.7778	-4.00082
C	-0.01439	1.88123	-3.15819
C	0.097	3.23384	-3.56178
C	0.06401	4.0295	-2.44706

C	-0.07165	3.20739	-1.2966
C	-0.10228	3.71801	0.
C	-0.07165	3.20739	1.2966
C	0.06401	4.0295	2.44706
C	0.097	3.23384	3.56178
C	-0.01439	1.88123	3.15819
C	-0.01484	0.7778	4.00082
N	0.13474	-1.04826	2.37625
N	0.13474	-1.04826	-2.37625
N	-0.08926	1.89534	-1.75771
N	-0.08926	1.89534	1.75771
C	0.0133	-4.40653	0.6871
C	0.0133	-4.40653	-0.6871
H	-0.03485	1.00672	-5.06426
H	-0.08031	4.80733	0.
H	-0.03485	1.00672	5.06426
H	0.45434	-0.55536	1.55436
H	0.45434	-0.55536	-1.55436
H	-0.53496	1.14549	-1.2486
H	-0.53496	1.14549	1.2486
H	0.22362	3.54386	4.59196
H	0.15596	5.10794	2.40222
H	-0.32364	-1.6836	5.54871
H	-0.25943	-3.87415	3.97939
H	-0.06274	-5.26011	1.35123
H	-0.06274	-5.26011	-1.35123
H	-0.25943	-3.87415	-3.97939
H	-0.32364	-1.6836	-5.54871
H	0.22362	3.54386	-4.59196
H	0.15596	5.10794	-2.40222

6) Bis(pentafluorophenyl)-[16]nocorrole

C	-7.57527	0.01013	0.0256
C	2.95571	-2.34355	-1.02992
H	3.94009	-2.56426	-1.42521
C	-6.86842	-0.93561	0.75633
C	7.57547	-0.012	-0.01768
C	-5.48088	-0.94412	0.71118
C	2.97579	2.47627	0.87039
H	3.98615	2.79894	1.09461
C	1.828	3.19922	1.0534
H	1.74658	4.21294	1.42691
C	6.88819	-0.94406	0.74935
C	-6.89102	0.94216	-0.74418
C	-2.97638	-2.47602	-0.87288
H	-3.98703	-2.7998	-1.09417
C	6.87148	0.93483	-0.74977
C	5.48381	0.94438	-0.70874
C	-5.50388	0.9218	-0.77409
C	0.73234	-2.41346	-0.61128
C	5.501	-0.92262	0.77522
C	-4.76132	-0.02123	-0.05496
C	1.77867	-3.08094	-1.26268
H	1.68982	-3.97338	-1.86841

C	4.76121	0.02147	0.05457
C	-3.28791	-0.01408	-0.0831
C	-2.95513	2.34355	1.02666
H	-3.93899	2.5631	1.42387
C	-1.77805	3.0811	1.2588
H	-1.6886	3.97259	1.86586
C	-2.57771	-1.17795	-0.36323
C	2.61279	-1.22435	-0.24929
C	3.28777	0.01533	0.07829
C	-0.73904	-2.33916	-0.63683
C	-1.82864	-3.19901	-1.05611
H	-1.74758	-4.21371	-1.42701
C	2.5775	1.17957	0.35692
C	-0.73247	2.41503	0.60485
C	0.73886	2.34078	0.63013
C	-2.6129	1.22582	0.24363
N	1.20313	1.18117	0.22385
N	-1.29135	1.36966	-0.04303
N	1.29074	-1.36718	0.03546
N	-1.20304	-1.17854	-0.23324
H	-0.74091	0.60454	-0.42527
H	0.74009	-0.60149	0.41616
F	-4.85145	-1.84582	1.45006
F	-7.52027	-1.81536	1.5005
F	-8.89515	0.02204	0.06036
F	-7.56498	1.83579	-1.45082
F	-4.89237	1.81106	-1.54163
F	4.85735	1.84712	-1.44886
F	7.52618	1.81467	-1.49135
F	8.89542	-0.02494	-0.04854
F	7.55934	-1.83874	1.45735
F	4.88667	-1.81191	1.54047

7) Tetrakis(pentafluorophenyl)-[18]porphyrin

C	-4.23722	0.67606	-0.14655
C	-4.2378	-0.67238	-0.1468
N	-0.00089	-2.0926	0.00011
N	0.00102	2.09261	0.00032
N	2.01604	-0.00084	0.01968
C	-2.84323	1.07778	-0.0642
C	-2.84418	-1.07535	-0.06445
N	-2.01589	0.00085	-0.01959
C	-1.12362	-2.87467	-0.02146
C	-1.12106	2.8756	-0.02122
C	-0.68372	-4.23251	-0.01005
C	-0.68003	4.23308	-0.01006
C	0.68019	-4.23307	0.01032
C	0.68386	4.23252	0.01011
C	1.12118	-2.87558	0.02162
C	1.12375	2.87466	0.02158
C	2.84336	-1.07777	0.06444
C	2.84432	1.07534	0.06447
C	4.23737	-0.67605	0.1468
C	4.23797	0.67238	0.14672

C	-2.44084	2.42208	-0.05385
C	2.44314	2.41999	0.05439
C	-2.44299	-2.41998	-0.05436
C	2.44098	-2.42205	0.0544
H	-1.33967	-5.09291	-0.01306
H	-1.33529	5.09401	-0.01319
H	-5.08897	1.34155	-0.21047
H	-5.09014	-1.3371	-0.21095
H	1.33543	-5.09401	0.0135
H	1.33983	5.09292	0.01311
H	5.08912	-1.34155	0.21081
H	5.09032	1.33712	0.21071
H	0.00061	1.07575	0.00017
C	3.50136	3.46414	0.08315
H	-0.00047	-1.07575	-0.00025
C	3.73206	4.23656	1.22053
C	4.30919	3.70633	-1.0271
C	4.72044	5.21178	1.25737
C	5.30875	4.67042	-1.01098
C	5.51218	5.42643	0.13653
C	-3.49821	3.46723	-0.08256
C	-4.30601	3.70978	1.02762
C	-3.72807	4.24013	-1.21978
C	-5.30478	4.67469	1.01161
C	-4.71565	5.21617	-1.25651
C	-5.50739	5.43119	-0.13573
C	-3.50131	-3.46414	-0.08343
C	-4.30925	-3.70641	1.02672
C	-3.73195	-4.2364	-1.22092
C	-5.30887	-4.67043	1.01041
C	-4.7204	-5.21154	-1.25796
C	-5.51224	-5.4263	-0.1372
C	3.49826	-3.46714	0.08308
C	3.72831	-4.23996	1.2203
C	4.30578	-3.70994	-1.02727
C	4.71575	-5.21614	1.25692
C	5.30441	-4.675	-1.01138
C	5.50721	-5.43139	0.13599
F	-4.91674	-5.93004	-2.35278
F	-2.99534	-4.05278	-2.30893
F	-6.45764	-6.34933	-0.16245
F	-6.06162	-4.87826	2.07953
F	-4.13842	-3.00263	2.13729
F	-4.13589	3.0054	2.13791
F	-6.05744	4.88278	2.08074
F	-6.45196	6.35508	-0.16068
F	-4.91126	5.93528	-2.35106
F	-2.99149	4.05634	-2.30779
F	4.13548	-3.00573	-2.13764
F	6.05674	-4.88331	-2.0807
F	6.45165	-6.3554	0.16083
F	4.91149	-5.93514	2.35152
F	2.99198	-4.05595	2.30845
F	2.99548	4.05306	2.30858
F	4.91681	5.93043	2.35209
F	6.45753	6.34952	0.16157

F	6.0614	4.87817	-2.08019
F	4.1383	3.00243	-2.13758

8) Bis(pentafluorophenyl)-[20]orangarin

N	1.40726500	-0.74450500	-0.09952300
C	0.76224900	-1.89359500	-0.19751800
C	-0.68208600	-1.92274100	-0.29329900
C	-1.56438900	-2.91587500	-0.69195100
C	-2.85825500	-2.36951300	-0.65994900
C	-2.76144900	-1.04402800	-0.24344500
C	-3.80883900	-0.06072800	-0.10071200
C	-3.57915600	1.29351100	-0.10168800
C	-4.43998900	2.39732600	0.25535300
C	-3.70260200	3.54345300	0.27736200
C	-2.34733700	3.20297100	-0.06459200
C	-1.12002400	3.86250800	-0.06682200
N	-0.02746600	3.03923600	-0.20627100
C	1.03446700	3.81901500	-0.05609900
C	2.32049300	3.14883900	-0.03142900
C	3.66517200	3.53990500	0.03734800
C	4.43478100	2.37318800	0.05235900
C	3.55263400	1.27769600	0.00131300
C	3.77076100	-0.12588700	-0.01012300
C	2.75975600	-1.07452600	-0.06351700
N	2.29022700	1.80063900	-0.05174800
C	-0.71138000	5.21296100	0.15448700
N	-2.34380000	1.86750400	-0.32791400
C	0.66429600	5.18958300	0.15544100
N	-1.41653800	-0.79663900	-0.03290100
C	1.65329500	-3.02666600	-0.21959300
C	2.91002300	-2.50781200	-0.12687800
H	-1.01110500	-0.00847400	0.45673700
H	-5.48568800	2.29178800	0.51735300
H	-4.03976500	4.53572300	0.54744300
H	-1.35752700	6.07215600	0.29320100
H	1.33885400	6.02411400	0.30819600
H	4.02567200	4.55995900	0.06564100
H	5.51532200	2.30975500	0.09295600
H	3.84749000	-3.05113500	-0.10309500
H	1.36324900	-4.06889200	-0.28033300
H	-1.28570400	-3.91300700	-1.00718300
H	-3.77867500	-2.86152600	-0.94899000
H	1.45299800	1.21980900	-0.10312800
H	-1.50737600	1.43895100	-0.70226500
C	5.18301400	-0.58032600	0.01616400
C	5.77869200	-1.16635600	-1.09958700
C	5.97225500	-0.41665800	1.15359200
C	7.10448300	-1.58006800	-1.08715900
C	7.29991700	-0.82248400	1.18675800
C	7.86637600	-1.40575500	0.06073500
C	-5.19169800	-0.55701100	0.03088800
C	-5.54955100	-1.45020500	1.04617800
C	-6.19854000	-0.17785200	-0.86155700
C	-6.84032300	-1.94374900	1.16983500

C	-7.49728700	-0.65735400	-0.75154100
C	-7.81821100	-1.54474300	0.26695900
F	8.02552700	-0.66318600	2.28319500
F	5.45774700	0.12678500	2.24805900
F	9.12921000	-1.79577200	0.08267600
F	7.64700900	-2.13152000	-2.16219400
F	5.08447800	-1.33567100	-2.21613300
F	-5.93195600	0.65109700	-1.86269000
F	-8.42623600	-0.28314100	-1.61761200
F	-9.05109700	-2.00501600	0.38024700
F	-7.14971300	-2.77736700	2.15054400
F	-4.65328700	-1.83232300	1.94369500

9) Tris(pentafluorophenyl)-[22]smaragdyrin

N	2.73888	1.29661	-0.11418
C	2.45335	2.59741	-0.22644
C	1.1206	3.06964	-0.29791
C	0.62243	4.34659	-0.57688
C	-0.76182	4.28757	-0.51044
C	-1.13369	2.97183	-0.17792
C	-2.43613	2.4714	0.06824
C	-2.81375	1.14857	0.23119
C	-4.08852	0.68584	0.66543
C	-4.09838	-0.6764	0.63884
C	-2.83061	-1.13866	0.18488
C	-2.46944	-2.45756	-0.03515
C	-1.16993	-2.95834	-0.29947
C	-0.80575	-4.24568	-0.72048
C	0.58096	-4.31926	-0.7586
C	1.08882	-3.07953	-0.36822
C	2.4334	-2.65812	-0.25696
C	3.61644	-3.42544	-0.26094
C	4.68219	-2.55226	-0.13017
C	4.16217	-1.24203	-0.05188
C	4.80634	0.01145	-0.0178
C	4.11295	1.22093	-0.06457
C	9.099	-0.0139	0.08691
C	8.43522	0.44344	-1.04379
C	7.04654	0.44709	-1.07025
C	6.28754	0.01295	0.01664
C	6.98383	-0.44212	1.13645
C	8.37156	-0.46034	1.18243
C	4.68505	2.54524	-0.12317
H	5.74115	2.78438	-0.08312
C	3.6425	3.41144	-0.24007
H	3.67804	4.4922	-0.30802
H	1.22788	5.20791	-0.8281
H	-1.45787	5.09506	-0.69794
C	-3.51686	3.48558	0.14437
C	-3.57601	4.42226	1.17765
C	-4.57585	5.38424	1.24124
C	-5.55571	5.42132	0.25722
H	1.18258	-5.16192	-1.07443

C	-5.52742	4.50132	-0.78244
C	-4.51374	3.55342	-0.83024
H	-4.88882	1.33541	0.99623
H	-4.90775	-1.32738	0.94374
C	-3.55465	-3.46762	0.00729
C	-3.59649	-4.45318	0.9955
C	-4.59578	-5.4165	1.03226
C	-5.59441	-5.40572	0.06668
C	-5.58494	-4.43627	-0.92748
C	-4.57093	-3.48737	-0.94967
H	-1.50548	-5.02578	-0.9916
H	3.65743	-4.50483	-0.33147
H	5.73378	-2.80856	-0.10037
N	0.03572	2.24627	-0.09217
H	0.12799	1.33903	0.34215
N	0.0074	-2.24712	-0.13288
H	0.05806	-1.42694	0.45763
N	2.79328	-1.3548	-0.11042
H	2.24341	-0.49111	-0.18436
N	-2.02684	0.00304	0.0097
H	-1.35576	0.0113	-0.752
F	9.00383	-0.89278	2.26341
F	6.32082	-0.869	2.2035
F	10.42082	-0.02492	0.12009
F	9.12918	0.86041	-2.09252
F	6.44942	0.87255	-2.17544
F	-2.6663	4.40733	2.14222
F	-4.60892	6.25672	2.23755
F	-6.5144	6.3299	0.31133
F	-6.45832	4.53979	-1.72408
F	-4.51347	2.69964	-1.8455
F	-2.66967	-4.48475	1.94348
F	-4.61066	-6.33542	1.98635
F	-6.55287	-6.31564	0.0954
F	-6.53348	-4.42946	-1.85211
F	-4.58867	-2.58616	-1.92284

10) Tris(pentafluorophenyl)-[22]isosmaragdyrin

N	-2.78404600	-1.35794600	0.08455100
C	-2.41805900	-2.65678100	0.37880400
C	-1.08695700	-3.08542200	0.31388300
C	-0.59457800	-4.40649700	0.33047400
C	0.77268900	-4.35141900	0.18964300
C	1.15591000	-2.98876500	0.10553300
C	2.44560800	-2.44818900	0.04946800
C	2.79564700	-1.08750400	0.04018000
C	4.18398800	-0.67766300	0.11193900
C	4.18377500	0.67025000	0.12510800
C	2.79662400	1.08120200	0.04294000
C	2.44633500	2.44049100	-0.00704200
C	1.15844900	2.97658600	-0.12224500
C	0.77942000	4.33449200	-0.27290200
C	-0.58578700	4.38491000	-0.43465700
C	-1.08027800	3.06599600	-0.37225400
C	-2.41075500	2.63696900	-0.44583900

C	-3.56823900	3.30841400	-0.87670200
C	-4.63266300	2.42883300	-0.78993200
C	-4.15860000	1.19372800	-0.29914900
C	-4.83477400	-0.00736800	-0.03512600
C	-4.16180400	-1.20929000	0.23300600
C	-9.12849200	-0.00915900	-0.06737600
C	-8.42485900	-0.84854900	-0.92118300
C	-7.03670100	-0.83544900	-0.90732200
C	-6.30990800	-0.00777100	-0.04649100
C	-7.05012300	0.81982000	0.80291700
C	-8.43838600	0.83117400	0.79659200
C	-4.64345800	-2.45138200	0.69856400
H	-5.67083600	-2.64587100	0.97967400
C	-3.58162300	-3.33435400	0.78265400
H	-3.61018200	-4.35307000	1.14825900
H	-1.21420000	-5.29146700	0.40093200
H	1.45732700	-5.18816500	0.14421800
C	3.54876900	-3.44633900	0.03469000
C	3.89322000	-4.17159300	1.17422900
C	4.91563700	-5.11189000	1.16722800
C	5.62908100	-5.33816200	-0.00254500
H	-1.20231700	5.26685700	-0.55352300
C	5.31347300	-4.62824200	-1.15374300
C	4.28149900	-3.69900000	-1.12421900
H	5.03879400	-1.33963800	0.16935500
H	5.03865100	1.33126600	0.19054300
C	3.54130400	3.44700900	0.02954300
C	3.76184500	4.23230100	1.16001500
C	4.76786800	5.18902400	1.20724400
C	5.58724300	5.37516500	0.10165900
C	5.39243100	4.60950000	-1.04063900
C	4.37378200	3.66558900	-1.06724300
H	1.46622300	5.17073500	-0.26692000
H	-3.59122500	4.32059100	-1.26035700
H	-5.65588700	2.61954000	-1.08828700
N	-0.00262400	-2.24907800	0.15513600
H	0.04822700	-1.23863000	0.24519200
N	0.00043000	2.23548300	-0.16344400
H	0.05577500	1.22291200	-0.22793400
N	-2.78285700	1.34404500	-0.13260300
H	-2.29686500	0.82046800	0.58572800
N	1.95999800	-0.00308900	0.00433200
F	-9.10771200	1.62436400	1.61971800
F	-6.43133500	1.62029700	1.66142900
F	-10.45022900	-0.01000400	-0.07704900
F	-9.08109300	-1.64231800	-1.75423600
F	-6.40482200	-1.63480400	-1.75735900
F	3.23687900	-3.97617600	2.31119000
F	5.21908800	-5.78663000	2.26685500
F	6.60699100	-6.22866400	-0.02072000
F	5.99235700	-4.84749000	-2.27038300
F	4.00618500	-3.03887900	-2.24094300
F	2.99572400	4.08168000	2.23320400
F	4.95367400	5.91934400	2.29742100
F	6.55056600	6.28096800	0.13587300
F	6.17066500	4.79195100	-2.09753600

F	4.21152600	2.95713800	-2.17673200
H	-2.28727500	-0.82240100	-0.61748500

11) [16]porphyrin

C	2.85188700	-0.96679200	0.02911000
N	2.05046300	0.05369000	-0.14461700
C	2.82395300	1.17528300	0.13869000
C	2.34232700	2.44618200	0.16004300
C	0.96678800	2.85187000	-0.02911100
N	-0.05369400	2.05045300	0.14463900
C	-1.17528500	2.82394400	-0.13868400
C	-2.44618800	2.34232900	-0.16003400
C	-2.85188600	0.96679200	0.02911000
N	-2.05046300	-0.05369000	-0.14461700
C	-2.82395300	-1.17528300	0.13869000
C	-2.34232700	-2.44618200	0.16004200
C	-0.96678800	-2.85187000	-0.02911100
N	0.05369400	-2.05045300	0.14463900
C	1.17528500	-2.82394400	-0.13868400
C	2.44618800	-2.34232900	-0.16003400
C	4.21904100	-0.56168200	0.39311600
C	4.19950300	0.78621600	0.44500700
C	0.56168500	4.21902700	-0.39310700
C	-0.78621200	4.19948100	-0.44504700
C	-4.21904100	0.56168200	0.39311400
C	-4.19950300	-0.78621600	0.44500800
C	-0.56168500	-4.21902700	-0.39310700
C	0.78621200	-4.19948100	-0.44504700
H	-5.05229900	1.23141000	0.57736400
H	-5.00494400	-1.46613800	0.69867000
H	-3.06366400	-3.24744000	0.32962100
H	-1.23141900	-5.05228100	-0.57735900
H	1.46612900	-5.00491700	-0.69873700
H	3.24744000	-3.06367600	-0.32960600
H	5.05229800	-1.23141100	0.57736700
H	5.00494500	1.46613800	0.69866700
H	3.06366400	3.24744000	0.32962100
H	1.23141900	5.05228100	-0.57735900
H	-1.46612900	5.00491700	-0.69873700
H	-3.24743900	3.06367600	-0.32960700

12) [20]porphyrin

C	-2.96630800	1.09285400	0.04858200
C	-4.28369400	0.64799000	0.03208600
C	-4.26536700	-0.75533500	-0.03065900
C	-2.93690500	-1.16577600	-0.04963600
C	-2.40966800	-2.49733700	-0.04698400
C	-1.11508300	-2.89544700	-0.07094500
C	-0.62214700	-4.23803500	0.12320900
C	0.72849100	-4.22153600	0.12384500
C	1.18861500	-2.86725300	-0.06998400
C	2.47299700	-2.43741800	-0.04529900
C	2.96635700	-1.09289400	-0.04840300
C	4.28372700	-0.64799400	-0.03229600

C	4.26537000	0.75535500	0.03009700
C	2.93691500	1.16576400	0.04928600
C	2.40964900	2.49730600	0.04660500
C	1.11509100	2.89545200	0.07077800
C	0.62212000	4.23805200	-0.12315400
C	-0.72851500	4.22159900	-0.12343900
C	-1.18866800	2.86732600	0.07027800
C	-2.47300700	2.43739100	0.04573300
N	-2.16694800	-0.02624000	-0.00109700
N	0.02655900	-2.04323500	-0.20688800
N	2.16696500	0.02617900	0.00132200
N	-0.02656000	2.04320900	0.20695000
H	5.11706900	1.42396600	0.04876800
H	-5.15254600	1.29407900	0.05309600
H	-1.39721000	5.05694500	-0.29692100
H	1.27032400	5.08947100	-0.29629900
H	-5.11706800	-1.42393700	-0.04960500
H	-1.27038500	-5.08943500	0.29630300
H	1.39720400	-5.05681400	0.29758000
H	5.15261000	-1.29404000	-0.05332700
H	-1.15865500	-0.01234400	-0.00388600
H	0.02057400	-1.50912200	-1.07379500
H	1.15867200	0.01231000	0.00388800
H	-0.02040200	1.50927900	1.07397600
H	-3.23670700	3.20953800	-0.02735900
H	-3.15387300	-3.28832300	0.02591100
H	3.23659800	-3.20963200	0.02805700
H	3.15384800	3.28827800	-0.02659300

13) [18]porphycene

C	2.91824700	-1.33876200	0.00000700
C	3.19170900	-2.74045600	-0.00008900
C	1.98896900	-3.40476100	0.00004200
C	0.95975600	-2.42144100	0.00014100
C	-0.44244800	-2.54124400	0.00003000
N	-1.22593200	-1.44542300	-0.00026700
C	-2.50676800	-1.88777900	-0.00017000
C	-3.66612700	-1.08946800	-0.00026500
C	-3.84615900	0.28679100	-0.00019500
C	-2.91824600	1.33879000	-0.00015800
C	-3.19173500	2.74041200	0.00023300
C	-1.98897000	3.40473800	0.00038800
C	-0.95977400	2.42145100	-0.00009000
C	0.44243600	2.54122100	0.00001000
N	1.22594300	1.44539600	0.00002400
C	2.50682800	1.88778700	-0.00003100
C	3.66613700	1.08953700	-0.00007700
C	3.84613500	-0.28676800	-0.00005300
N	1.56304100	-1.20413800	0.00016000
C	-1.23178900	-3.74758500	0.00034200
C	-2.52974800	-3.33673300	0.00014100
N	-1.56306500	1.20412300	-0.00031000
C	1.23181200	3.74760000	0.00008800
C	2.52975900	3.33673100	-0.00004300

H	1.11682400	-0.26357800	0.00018800
H	-0.84906400	-4.76157100	0.00063800
H	1.82566500	-4.47526800	0.00006500
H	-3.42457500	-3.94932800	0.00038800
H	4.18539300	-3.17199700	-0.00021700
H	-4.59003100	-1.66861800	-0.00018400
H	4.88207100	-0.62462500	-0.00018400
H	-4.88208400	0.62466200	-0.00001300
H	4.59005100	1.66866600	-0.00018200
H	-1.82569800	4.47524900	0.00067500
H	0.84905600	4.76157200	0.00008700
H	-4.18541300	3.17196100	0.00050100
H	3.42461100	3.94928600	-0.00008300
H	-1.11686300	0.26352400	-0.00044000

14) [22]sapphyrin

C	-0.79720000	-3.56946200	0.00000000
C	-1.73796000	-4.63907000	0.00000000
C	-3.00105800	-4.10920600	0.00000000
C	-2.87371600	-2.69963500	0.00000000
C	-3.88969400	-1.75244700	0.00000000
C	-3.72584700	-0.37460900	0.00000000
N	-2.51358800	0.26340100	0.00000000
C	-2.82131100	1.57077800	0.00000000
C	-1.87189100	2.61416800	0.00000000
C	-2.12332900	4.00671200	0.00000000
C	-0.90975100	4.65449800	0.00000000
C	0.10710000	3.67021500	0.00000000
C	1.48286900	3.87030800	0.00000000
C	2.49017300	2.91619100	0.00000000
N	2.33753000	1.55454200	0.00000000
C	3.60420100	1.07944500	0.00000000
C	4.03389100	-0.25451800	0.00000000
C	3.35258600	-1.45407700	0.00000000
C	3.99063600	-2.72905500	0.00000000
C	3.02589900	-3.68695000	0.00000000
C	1.74423900	-3.05105600	0.00000000
C	0.58041900	-3.80226300	0.00000000
C	-4.24801700	1.80103600	0.00000000
C	-4.81679700	0.56879800	0.00000000
N	-1.52501800	-2.40787700	0.00000000
N	-0.51759600	2.44732900	0.00000000
C	3.88608600	3.30080000	0.00000000
C	4.58799800	2.14673700	0.00000000
N	1.99006800	-1.69044600	0.00000000
H	-1.45395500	-5.68437000	0.00000000
H	-3.94676500	-4.63661000	0.00000000
H	-4.74139700	2.76553900	0.00000000
H	-5.86977800	0.31061600	0.00000000
H	-4.90697100	-2.13989900	0.00000000
H	-0.72587600	5.72169500	0.00000000
H	-3.10773300	4.45591100	0.00000000
H	1.80160300	4.91160200	0.00000000
H	4.24991800	4.32219100	0.00000000

H	5.66260300	2.00160300	0.00000000
H	5.11618900	-0.38006700	0.00000000
H	3.15236900	-4.76268700	0.00000000
H	5.06551600	-2.86061200	0.00000000
H	0.79973300	-4.86912500	0.00000000
H	0.00000000	1.57464000	0.00000000
H	1.32203600	-0.93222300	0.00000000
H	-1.23442100	-1.43086900	0.00000000
